# Dual-Functioning Antibacterial Eugenol-Derived Plasticizers for Polylactide

**DOI:** 10.3390/biom10071077

**Published:** 2020-07-20

**Authors:** Wenxiang Xuan, Karin Odelius, Minna Hakkarainen

**Affiliations:** Department of Fibre and Polymer Technology, School of Engineering Sciences in Chemistry, Biotechnology and Health, KTH Royal Institute of Technology, Teknikringen 56, 100 44 Stockholm, Sweden; wxuan@kth.se (W.X.); hoem@kth.se (K.O.)

**Keywords:** plasticizer, polylactide, eugenol, levulinic acid, antibacterial

## Abstract

Dual-functioning additives with plasticizing and antibacterial functions were designed by exploiting the natural aromatic compound eugenol and green platform chemical levulinic acid or valeric acid that can be produced from biobased resources. One-pot synthesis methodology was utilized to create three ester-rich plasticizers. The plasticizers were thoroughly characterized by several nuclear magnetic resonance techniques (^1^H NMR, ^13^C NMR, ^31^P NMR, HSQC, COSY, HMBC) and by electrospray ionization-mass spectrometry (ESI-MS) and their performances, as plasticizers for polylactide (PLA), were evaluated. The eugenyl valerate was equipped with a strong capability to depress the glass transition temperature (*T*_g_) of PLA. Incorporating 30 wt% plasticizer led to a reduction of the *T*_g_ by 43 °C. This was also reflected by a remarkable change in mechanical properties, illustrated by a strain at break of 560%, almost 110 times the strain for the breaking of neat PLA. The two eugenyl levulinates also led to PLA with significantly increased strain at breaking. The eugenyl levulinates portrayed higher thermal stabilities than eugenyl valerate, both neat and in PLA blends. The different concentrations of phenol, carboxyl and alcohol functional groups in the three plasticizers caused different bactericidal activities. The eugenyl levulinate with the highest phenol-, carboxyl- and alcohol group content significantly inhibited the growth of *Staphylococcus aureus* and *Escherichia coli*, while the other two plasticizers could only inhibit the growth of *Staphylococcus aureus*. Thus, the utilization of eugenol as a building block in plasticizer design for PLA illustrated an interesting potential for production of additives with dual functions, being both plasticizers and antibacterial agents.

## 1. Introduction

The increasing demand for food, due to the increase in world population and the long-distance logistic chains, has prompted amplified requirements of food freshness. A prolonged shelf-life is highly desired if the nutrition of the food is not to be a trade-off. Innovative materials with delicately designed functions and targeting food packaging could provide solutions. Green building blocks that can be achieved from renewable biomass conversions in a commercial scale should be utilized in the design of the high-performance materials. If agricultural and food waste is used as converted biomass, green features of the building blocks will be further accentuated, contributing to a more sustainable engineering production for the society.

Polylactide (PLA), as a biodegradable replacement for commodity plastics, has been widely studied in the applications of packaging and has demonstrated the capability of maintaining food quality and light protection [1,2,3,4,5,6]. The U.S. Food and Drug Administration (FDA) has approved PLA to be used in food-contact packaging [7], therefore, the commercial utilization of PLA packaging has been increasingly realized [8]. To extend the shelf-life of food products, the microbiological contaminations inside the packaging need to be inhibited or minimized. Incorporating antimicrobial agents in the packaging is the simplest and most feasible means to achieve that aim and those agents can either be present as independent devices or be part of the packaging material itself [9]. Many antimicrobial agents have been evaluated as additives in PLA to examine the final antimicrobial performances, including catechin [10], nisin [11,12], copper nanoparticles [13], silver-based nanoclay [14], preservatives [15], lauric arginate [16], essential oil [17], thymol [18], natamycin [19], triclosan [20] and chitosan [21].

However, with the addition of antimicrobial agents, the mechanical and thermal properties of PLA are unavoidably altered. This can be critical for a packaging material, because PLA is generally a rigid and semicrystalline polymer and usually requires processing aids for flexible applications. Plasticizers are chemical additives that lower the glass transition temperature (*T*_g_) of plastics and improve ductility for easier processing. When plasticizer is blended with polymer, the chain mobility is facilitated, leading to decreased *T*_g_. As a result, other properties of the polymer that relate to chain mobility can be influenced, such as the crystallization process of polymer, which plays a critical role for the mechanical and thermal behaviors of polymer [22]. The performance of the plasticizer depends on several factors, including the molecular weight of plasticizer, chemical structure (e.g., balance between polar and nonpolar groups), linear or branched shape, flexibility of plasticizer chain and amount added [23]. Both the molecular weight and chemical structure influence the miscibility of the plasticizer with the polymer. Polar groups, such as ester groups, are usually required for forming strong secondary interactions between plasticizer and the polymer, while nonpolar groups often enhance the plasticization. Miscibility is desired for obtaining homogeneous and migration resistant polymer blends with reduced *T_g_*. Plasticizers with a lower molecular weight often have higher plasticizing efficiency but lower migration-resistance.

Several PLA plasticizers have been designed in previous works with various purposes, for instance for increased migration-resistance [24,25,26] or from renewable starting materials [24,27,28]. Citrates that can be produced from biobased starting materials, for example acetyl triethyl citrate and triethyl citrate, are recommended by the FDA to be used as plasticizers in food-packaging material [29]. In addition, the alkylsulfonic acid ester of phenol, another FDA-approved food-contact plasticizer [30], indicated the potential for the utilization of versatile building blocks in plasticizer design. This includes phenolic compounds that simultaneously also can act as antioxidants [31,32] and biocompatibilizers [33] in polymeric packaging materials. Nevertheless, some phenolic compounds have demonstrated cytotoxicity on mammalian cells and have aroused health concerns about their uses [34,35,36]. In addition to the mammalian toxicity, the biodegradation of plasticizers is another important issue when evaluating the full life-cycle of plastics. Nonbiodegradable plasticizers can create environmental contaminations and lead to problematic bioaccumulation [37,38,39,40]. Thus, comprehensive evaluations including toxicity and biodegradation tests are essential to ensure the plasticizers have no negative effect on human health and the environment.

Plasticizers with dual functions including plasticization ability and antibacterial properties would be a promising solution for flexible PLA packaging, providing longer shelf-life for the food. To achieve that goal, the realization of the benefits should be reflected in the considerations of plasticizer design. Levulinic acid, a green platform chemical, has demonstrated great miscibility and plasticization of PLA in its ester forms [28]. Valeric acid that can be produced by biomass conversion is also a good candidate to be utilized in plasticizer design since pentaerythritol tetravalerate has been approved by the FDA for use as a food-contact plasticizer [30]. Furthermore, eugenol, a natural phenolic compound exhibiting antimicrobial properties [41], has illustrated good potential in the development of high-performance materials [42]. Therefore, we anticipated that plasticizers designed from eugenol, levulinic acid and valeric acid, could have good plasticizing effects on PLA, while also providing antibacterial protections.

## 2. Materials and Methods

### 2.1. Materials

Levulinic acid (LeA; 97%), valeric acid (VaA; 99%), eugenol (99%) and *p*-toluene sulfonic acid monohydrate (PTSA; 98.5%) for the synthesis of plasticizers were purchased from Sigma-Aldrich and were not produced in a biobased way. Potassium carbonate (ACS grade; Arcos) and ethyl acetate (analytical reagent grade; Fisher Scientific) were utilized to remove unreacted eugenol and levulinic or valeric acids and to extract the plasticizers. N, *N*-Dimethylformamide (DMF; 99.8%; VWR) and pyridine (99.7%; VWR) were employed in ^31^P nuclear magnetic resonance (NMR) spectroscopy analyses in which *N*-hydroxy-5-norbornene-2,3-dicarboxylic acid imide (NHND; 97%; Sigma-Aldrich) and chromium (III) acetylacetonate (99.99% trace metal basis; Sigma-Aldrich) were incorporated as an internal standard and relaxation reagent, respectively. The derivatizing reagent was 2-chloro-4,4,5,5-tetramethyl-1,3,2-dioxaphospholane (TMDP; 95%; Sigma-Aldrich). Chloroform-d (CDCl_3_; 99.8%), from Cambridge Isotope Laboratories, was used in all NMR analyses. Dichloromethane (HPLC grade; Fisher Scientific) and polylactide (5200D; NatureWorks; M_n_ = 118,000 g/mol, Đ = 1.7) were used to prepare polylactide blends with or without plasticizers by solution casting. Methanol (hypergrade for LC-MS; Merck) served as solvent for electrospray ionization-mass spectrometry (ESI-MS) analysis. Regarding antibacterial evaluation, LB broth and agar were provided by Sigma-Aldrich and qualitative filter papers and ethanol absolute were purchased from VWR. All chemicals were used as received.

### 2.2. Synthesis of Plasticizers

Two eugenyl levulinates (TL and ML) were synthesized through reactions between LeA and eugenol. TL was prepared in a molar ratio of 3:1 for eugenol:LeA and ML was prepared in an equal molar ratio (1:1). LeA (3.5 g, 0.03 mol) and eugenol (14.8 g, 0.09 mol) were used to synthesize TL and LeA (11.6 g, 0.1 mol) and eugenol (16.4 g, 0.1 mol) to generate ML. An eugenyl valerate (MV) was derived from VaA (5.1 g, 0.05 mol) and eugenol (8.21 g, 0.05 mol). In addition, eugenol was reacted alone with catalyst as a control experiment (8.21 g, 0.05 mol). All reactions were catalyzed by 1 mol% PTSA equivalent to eugenol. The reagents were stirred and reacted in a 250 mL three-necked round-bottomed flask at 140 °C for 24 h under nitrogen atmosphere. The flask was connected to a Liebig condenser equipped with a 25 mL round-bottomed flask to remove and collect the formed water during reaction. At the end of the reaction time, the reaction liquor was cooled to 22 °C and mixed with 20 mL water with vigorous stirring. Then, 20 wt% K_2_CO_3_ (aq.) was added into the mixture with vigorous stirring until pH > 12. The plasticizers in alkaline mixture were later extracted by ethyl acetate in a separation funnel. The desired ethyl acetate phase was separated and further purified with K_2_CO_3_ dilution (pH > 12) twice to remove eugenol residue completely, followed by three repetitions of an extraction process with deionized water to remove alkali residue. The plasticizers were recovered by rotary evaporation of ethyl acetate at reduced pressure.

### 2.3. Nuclear Magnetic Resonance (NMR)

The main linkages in the synthesized plasticizers were determined by 1D-NMR (^1^H NMR, ^13^C NMR, ^31^P NMR) and 2D-NMR (HSQC, COSY, HMBC). All analyses were completed by a Bruker Avance 400 spectrometer at 25 °C. The frequency of the equipment was 400, 100 and 162 MHz for ^1^H NMR, ^13^C NMR and ^31^P NMR respectively. Two scans, 16 dummy scans and a relaxation time of 1.5 s were applied in HSQC and 4 scans, 8 dummy scans and a relaxation time of 2 s were used for COSY. Regarding HMBC, 4 scans, 16 dummy scans and a relaxation time of 1.5 s were utilized. The chloroform residue in chloroform-d was utilized as an internal reference to calibrate all the spectra (except ^31^P NMR) and phase corrections on both directions were processed on HSQC and COSY spectra. The procedures of ^31^P NMR analyses were described individually. In the preparation of each ^31^P NMR sample, around 30 mg synthesized plasticizers was mixed in sequence with 100 μL DMF, 100 μL pyridine, 50 μL stock solution of internal standard (60 mg/mL NHND and 10 mg/mL chromium acetylacetonate in pyridine) and 400 μL chloroform-d in a glass vial. Next, 100 μL TMDP was added into the vial and the mixture was left on an orbital shaker for 30 min to react. Then, ^31^P NMR analyses were conducted with following parameters—256 scans, 4 dummy scans and a relaxation time of 6 s. The peak from water reacted with TMDP (δ_P_ = 132.2 ppm) was taken as the internal reference. All data were processed by MestReNova v9.0.0 software.

### 2.4. Electrospray Ionization Mass Spectrometry (ESI-MS)

The synthesized plasticizers were analyzed by a Finnigan LCQ ion trap mass spectrometer in positive mode. The plasticizers were diluted in methanol to a concentration below 0.1 mM. The diluted solution was pumped in by a syringe with a speed of 25 μL/min and the ion source was set to 4.5 kV. The capillary temperature was adjusted to 200 °C. The nebulizing gas was nitrogen.

### 2.5. Preparation of PLA Films

The neat PLA film and plasticized PLA films were obtained by solution casting in petri dishes with a diameter of 186 mm. The granular PLA was dissolved in 100 mL dichloromethane either alone or with 10, 20 or 30 wt% plasticizers (in total weight) to produce neat and plasticized PLA films. The total weight of polymer or polymer-plasticizer set was 4.0 g. The polymer solution was stirred at 22 °C for at least 1.5 h before pouring into petri dishes. The petri dish was kept in a fume hood at 22 °C for at least 3 days and later the film was removed from the petri dish. The remaining solvent traces in the films were removed in a drying chamber at 25 °C with reduced pressure for at least 4 days. The thickness of a typical film was 151 ± 19 μm. The plasticized PLA films were denoted as “weight fraction of plasticizer + abbreviation of plasticizer name” (e.g., 30 MV represented the PLA film that had been plasticized by 30% MV plasticizer). The neat PLA film was referred to as PLA100.

### 2.6. Differential Scanning Calorimetry (DSC)

The glass transition temperatures (*T_g_*) of the neat and plasticized PLA films were assessed by Mettler Toledo DSC 820 Module in nitrogen atmosphere. The DSC program consisted of a scan cycle (25–200 °C, kept for 2 min→ −30 °C, kept for 2 min) to remove thermal history and a following second heating scan (−30–200 °C). The cooling and heating rates were set to 10 °C per minute and the midpoint of glass transition in the second heating scan was extracted as the *T_g_*. Triplicate samples were analyzed.

### 2.7. Thermal Gravimetric Analysis (TGA)

The thermal stabilities of the neat plasticizers and plasticized PLA films were measured by using a Mettler Toledo TGA/DSC 851e module instrument. The elevating heating scan was 25 °C to 500 °C with a heating rate of 5 °C per second. Nitrogen atmosphere was provided by a 50 mL/min nitrogen flow. Triplicate samples were analyzed. The onset temperature corresponding to weight loss of 5% was taken for comparison.

### 2.8. Tensile Test

The mechanical performances of neat and plasticized PLA films were evaluated by tensile test on INSTRON 5944 module equipped with pneumatic grips. The PLA films were cut into rectangular specimens with a constant width of 5 mm and a length of roughly 100 mm. The thickness of specimen was measured by digimatic indicator (ID-C112B, Mitutoyo Corp., Kawasaki, Japan) with resolution of 0.001 mm. The thickness values at two ends and middle position of the specimen were averaged as the mean thickness of that specimen. All specimens were conditioned before testing for 40 h at RH 50 ± 5% and 23 °C ± 1 °C, as required in ASTM D618-13 (Standard Practice for Conditioning Plastics for Testing). A load cell of maximum 500 N was set at a crosshead speed of 20 mm/min and the length between gauges was 20 mm. Data from at least 6 specimens were processed for each sample.

### 2.9. Zone of Inhibition Test

The potential migration and antibacterial effect of the synthesized plasticizers were examined by zone of inhibition test on *Staphylococcus aureus* (DSM 2569) and *Escherichia coli* (ORN 178). The PLA disks blended with plasticizers, filter paper disks loaded with plasticizers and the original plasticizers alone were assessed for antibacterial performances. All disks were of a diameter of 9 mm. To obtain filter paper loaded with 1 mg plasticizers, a dilution of plasticizers in 75 vol % ethanol with a concentration of 0.1 g/mL was prepared in prior to adding 10 μL dilution on a paper disk by pipette. This was repeated five times to prepare disks loaded with 5 mg plasticizers. The disks were subsequently dried thoroughly between each plasticizer addition. All control paper disks without plasticizers were treated with 75 vol % ethanol with the same preparation process as the disks loaded with plasticizers and dried completely before use. The bacteria were incubated in LB broth overnight and then diluted with saline for further use. A volume of 500 μL of bacteria dilution, equivalent to 8.5 × 10^5^ CFU *Staphylococcus aureus* or 3.5 × 10^5^ CFU *Escherichia coli*, was inoculated on each LB agar plate. The disks or neat plasticizers were placed on top of the inoculated agar plates. The petri dishes were incubated for at least 18 h at 37 °C before examination. The diameters of inhibition zones were measured by Cocraft^®^ vernier caliper.

## 3. Results & Discussion

Eugenol, an aromatic biobased substance with antibacterial properties, levulinic acid, an abundant green platform chemical [43], along with biobased valeric acid, are versatile building blocks that have high potential in the design of ecofriendly property-enhancing additives for PLA. A series of biobased ester-type compounds were, thus, synthesized based on these three starting chemicals, forming a variety of chemical bonds due to the diverse reactivities. The synthesized compounds were thoroughly characterized and their potential dual functions combining plasticization and introduction of antibacterial properties in PLA were examined.

### 3.1. Synthesis and Chemical Structure of the Plasticizer Candidates

**Figure 1:**
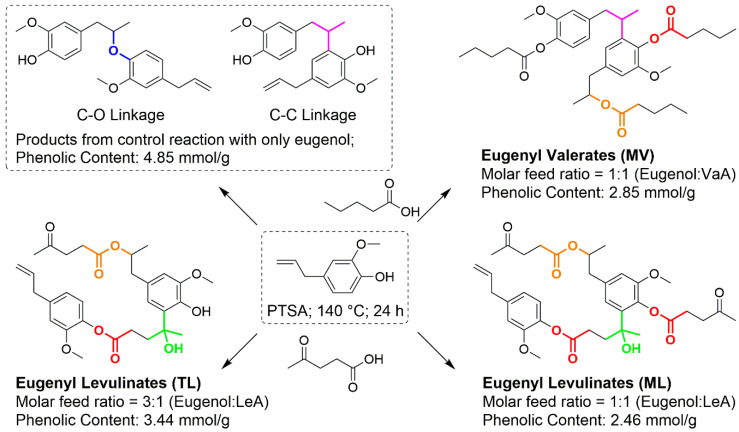
Schematic presentation of the synthesis, representative chemical bonds in the products, from a control reaction with eugenol only and the plasticizer candidates including eugenyl levulinates (ML and TL) and eugenyl valerate (MV) (C-O linkages in blue; C-C linkages in purple; phenolic ester bonds in red; aliphatic ester bonds in orange; converted-ketones in green).

Three plasticizer candidates that can be derived from biobased resources, two eugenyl levulinates (ML and TL) and eugenyl valerates (MV), with aliphatic and phenolic ester functionalities, were successfully synthesized from eugenol and levulinic acid or valeric acid under classic esterification conditions (protonic catalyst and high temperature). The yield for MV, ML and TL was 65.2%, 67.4% and 77.4%, respectively. All three plasticizer candidates were mixtures of esters with different molecular weights. This is because several types of reactions occurred simultaneously, including esterification, electrophilic aromatic substitution and nucleophilic addition, resulting in several different linkages in the plasticizer candidates (Figure 1). The C-O and C-C linkages in eugenol contributed to the formation of diverse products during synthesis of MV. Additional reactions took place during the synthesis of ML and TL as carbonyl carbons of the ketones from levulinic acid reacted with the eugenol rings by nucleophilic additions.

The versatile reactivity of eugenol and levulinic acid resulted in a broad distribution of reaction products as shown by the ESI-MS analysis presented in Figure 2. To further fingerprint the formed products, two-dimensional NMR, together with one-dimensional NMR, was utilized as an essential tool to examine the main linkages existing in the plasticizer candidates MV, ML and TL and in the product formed during a control reaction with only eugenol (Figures S1–S21). The peak integrations and assignments for one-dimensional NMR spectra, Tables S1 and S2, and peak assignments for ESI-MS, Tables S3–S6, jointly supported the structural assignment. The results of the analyses are presented in brief below, while more details are presented in Supporting Information.

**Figure 2:**
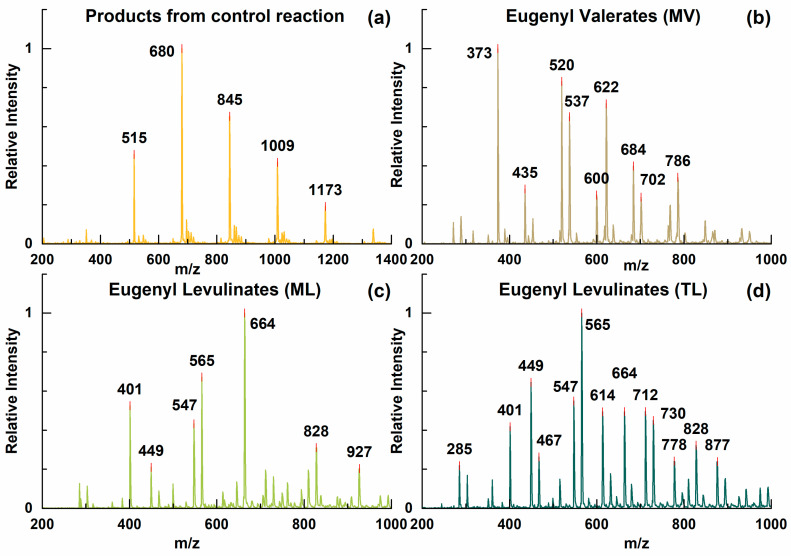
Electrospray ionization-mass spectra of products from a control reaction with only eugenol (**a**), eugenyl valerates-MV (**b**), eugenyl levulinates-ML (**c**) and eugenyl levulinates-TL (**d**).

In principle, two classes of chemical bonds were formed in the control reaction with eugenol only and they are C-O linkages and C-C linkages, as shown in Figure 1. Eugenol oligomers were generated by those two means, as seen in the ESI-MS spectrum (Figure 2a). The allyl group in eugenol could be converted into carbocation with the presence of PTSA at high temperature. Termination of the carbocation formed C-O linkages (or C-C linkages) between allyl and phenolic hydroxyl groups (or aromatic rings). When valeric acid reacted with eugenol, the carboxyl groups in the valeric acid molecules were directly added to the double bonds in the allyl groups or encountered dehydrations with phenolic hydroxyl groups, both reactions resulted in the formation of ester bonds that were presented in the plasticizer candidate MV. C-O linkages and C-C linkages were also found in MV, and as a result, compounds with several connected aromatic rings were found in the ESI-MS spectrum (Figure 2b). Similarly, the carboxyl groups in levulinic acid reacted with allyl groups of eugenols and subsequently generated aliphatic and phenolic ester bonds in the plasticizer candidates ML and TL. The ESI-MS spectrum (Figure 2c,d) suggested more diverse products in ML and TL. The ketone groups in levulinic acids were subjected to nucleophilic addition and hence developed another type of linkage between aromatic rings and levulinic acids, which contributed to an alternative pathway to connect eugenols together with previous C-O and C-C linkages. In addition, due to this pathway, levulinic acids could be bridged to eugenols directly and cause the presence of carboxylic acid signals in ^31^ P NMR characterizations (Figure S22 and Table S2).

### 3.2. Miscibility of PLA with Plasticizer Candidates

**Figure 3:**
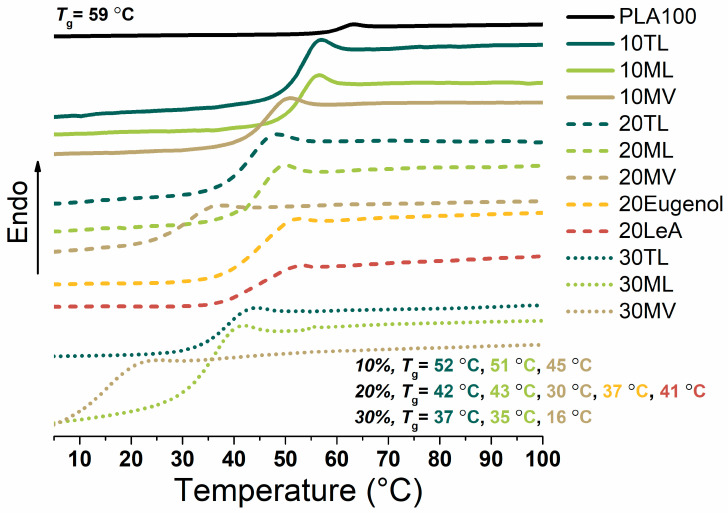
Differential scanning calorimetry (DSC) thermograms for neat PLA and the different blends.

PLA was blended with the plasticizer candidates (10, 20 and 30 wt%) and the starting compounds (eugenol and levulinic acid) were casted into films. The transparent blend films, and a neat PLA film, were characterized by DSC to examine the miscibility of the plasticizer candidates with PLA and to characterize the thermal properties of the blends. Decreased *T_g_* values for all the PLA blends were observed, as compared to neat PLA. No recrystallization and melting process was observed, i.e., the PLA itself and its blends were both amorphous. Only one *T_g_* in each DSC diagram was observed (Figure 3 and Table S7). A depression of the *T_g_* further developed with an increasing amount of plasticizer, indicating that the improvement of PLA chain mobility because of the presence of plasticizer candidates. All three synthesized plasticizer candidates TL, ML and MV were miscible with PLA within the whole measurement range as indicated by the decreasing *T_g_* values as a function of plasticizer concentration. The lowest *T_g_* (16 °C) was obtained when 30 wt% of plasticizer candidate MV was added, resulting in a *T_g_* below room temperature. The *T_g_* depressions for plasticizer candidate TL and ML were very close to each other at 10, 20 and 30 wt% concentrations. Although the PLA blends with 20 wt% neat eugenol or levulinic acid had similar *T_g_* values to PLA with 20 wt% TL or ML, the eugenol and levulinic acid blends portrayed phase separations after storage for a few weeks, i.e., eugenol and levulinic acids migrated from the materials bulk to the surface. The performances of the three plasticizer candidates were comparable to other naturally derived commercial plasticizers. For instance, PLA plasticized with 20 wt% commercial acetyl tributyl citrate (ATBC) [28] or di*-*2*-*etylhexyladipate (DOA) lowered the *T_g_* of PLA down to 23 °C and 40 °C, respectively [44]. As another example, a *T_g_* down to 28 °C was obtained by addition of 10 wt% oregano essential oil which was enriched with phenolic compounds [45]. Additionally, the *T_g_* of PLA decreased to 52 °C [46], 49 °C [47] and 52 °C [47] by the addition of 10 wt% oligoesters, epoxidized linseed oil and epoxidized soybean oil, respectively. Previously hexanoates were produced from liquefied wood flour, which depressed the *T_g_* of PLA to 25 °C with 10 wt% addition [27]. These hexanoates had a close M_w_ range comparable to TL, ML and MV, and they also contained ring structures and free hydroxyl groups from monosaccharides and disaccharides, which were similar to the eugenol structures in the three synthesized plasticizer candidates.

### 3.3. Thermal Stability of PLA Blends with Plasticizer Candidates

**Figure 4:**
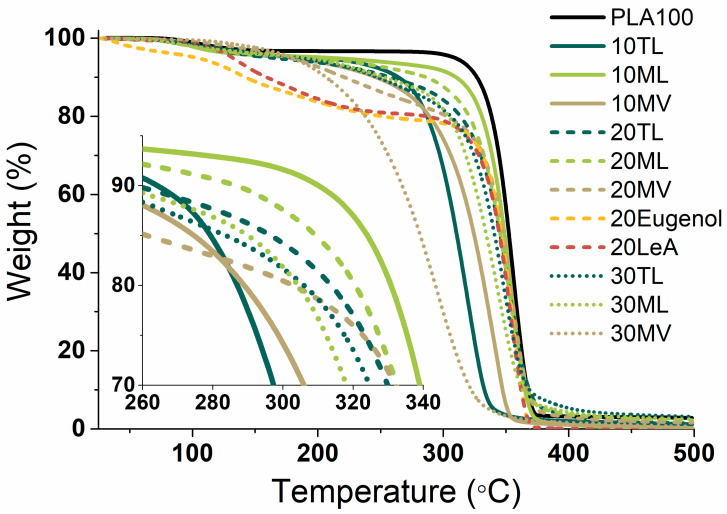
TGA curves of neat PLA and its blends.

PLA blends with the plasticizer candidates were further characterized by gravimetric analysis to assess the thermal stabilities of the blends which were indicated by the degradation temperatures where 5 wt% mass loss occurred (T5). The higher T5 temperature suggested better thermal stability of the blend. The results are illustrated in Figure 4 and Table S8. PLA blends with ML demonstrated higher thermal stability with higher T5 values as compared to the blends with TL and MV. With 20 or 30 wt% addition of the plasticizer candidates, PLA blends with ML and TL portrayed similar degradation curves. However, 10 ML demonstrated significantly higher thermal stability compared to that of 10 TL. The blend 10 MV had the lowest T5 value (179 °C), as compared to 207 °C for 10 ML and 198 °C for 10 TL. When the amount of plasticizer candidate MV increased to 30 wt%, the thermal stability of 30 MV significantly deteriorated, although its T5 value was still 182 °C, which could still allow melt-processing of the material. In addition, two stages of thermal degradation were observed in 20 Eugenol and 20 LeA (Figure 4), including the evaporation of eugenol and LeA in the first step and the degradation of PLA in the second step. That evaporation process had also been recorded in Figure S23 and Table S9.

### 3.4. Mechanical Performance of PLA Blends with Plasticizer Candidates

**Figure 5:**
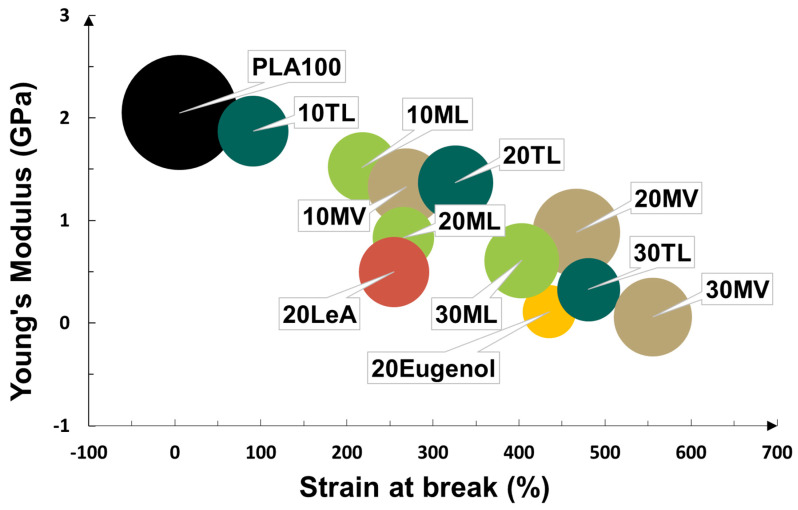
Mechanical properties of PLA blends (the relative bubble size symbolizes stress at break values).

The PLA blends were examined by tensile testing to determine the plasticizing effect of the three synthesized plasticizer candidates and to evaluate the mechanical properties after the addition of the plasticizer candidates. As expected, the mechanical properties of PLA were altered by the addition of plasticizer candidates, which all had good affinity to PLA and lowered the *T_g_* of PLA. A common trend for all PLA blends with increasing concentration of plasticizer candidates was that, due to the improved chain mobility, the Young’s modulus and tensile stress at break decreased while the tensile strain at break increased. This is demonstrated in Figure 5 and Figures S24–S35 and Table S10. The bubbles in Figure 5 symbolized the tensile stress at break of the blends and their values were reflected by the relative size of the bubbles, which were compared to that of neat PLA. The size of the bubble generally decreased after the addition of the plasticizer candidates. For 20 TL, as an example, the Young’s modulus, strain at break and stress at break were 1.4 GPa, 330% and 24 MPa respectively, whereas the values were 1.9 GPa, 91% and 21 MPa for 10 TL. In contrast to that, the PLA100 had a Young’s modulus of 2.1 GPa and a value of stress at break of 57 MPa, with a strain at break of 5.4% [28]. Hence, all PLA blends with the three different plasticizer candidates were plasticized. Although PLA blends with 20 wt% eugenol or levulinic acid suggested plasticization as well, their modulus was rather poor as compared to the performances of the eugenol and levulinic acid-derived plasticizer candidates. Furthermore, phase separation was observed in all PLA blends with 20% eugenol or 20% levulinic acid some days after tensile testing. Part of the films became rigid and the surface of the films became sticky, indicating migration of plasticizer to the surface.

Interestingly, the *T_g_* of the PLA blends plasticized by TL and ML indicated that they had a very similar ability to enhance the chain mobility of PLA, since the *T_g_* of their blends were very similar at the same plasticizer concentration. However, the plasticizer candidate TL resulted in higher strain at break compared to ML at 20 and 30 wt% addition. The levulinic acid content was significantly higher in ML plasticizer compared to TL, which led to lower strain at break. This agrees with the higher strain at break for the blend containing only 20 wt% eugenol compared to the blend containing only 20 wt% levulinic acid, i.e., eugenol was initially more effective plasticizer than levulinic acid. Although, it should be mentioned that the eugenol plasticized films were not stable with time and there were significant migration problems. Noticeably, addition of plasticizer candidate MV resulted in the largest improvement in strain at break at each concentration among the three plasticizer candidates. The dominant structure in MV had a lower molecular weight (Mw = 350) than the ones in TL (Mw = 641) and ML (Mw = 542). The smaller Mw for the plasticizer enables a higher diffusion rate in polymers and a higher number of plasticizer molecules when the same wt% of plasticizer is used, which usually leads to higher strain at break [23]. From the aspect of chemical structure, the balance between polar and nonpolar groups is important for the plasticization efficiency [37]. The MV plasticizer has the lowest level of hydroxyl and carbonyl groups in its structure as compared to TL and ML and ester bonds are the predominant linkages in it. This probably ensured more interactions with the ester groups in the PLA backbone. Furthermore, the TL plasticizer had a longer flexible nonpolar alkane chain, which is expected to improve the plastification. The higher amount of polar groups in TL and ML could also result in stronger secondary interactions between the plasticizer molecules, which could further lower the plasticizing efficiency due to the weaker affinity between PLA and plasticizer molecules [23].

With 20 wt% addition of ATBC, the plasticized PLA film demonstrated a Young’s modulus of 0.26 GPa and could be elongated to 640% with a stress of 49 MPa at break [28]. As compared to the blends with 20 wt% ATBC, the blends with 20 wt% eugenol-based plasticizer TL, ML and MV all had a higher Young’s modulus and their strain at break values were lower, ranging from 260 to 470%. This enables the possibility to tune mechanical properties based on the application requirements. Moreover, the 20 TL blend had an equal Young’s modulus and somewhat higher strain and stress at break than PLA blended with 20 wt% DOA, another commercial biobased plasticizer [44]. Therefore, the achieved mechanical performances of the PLA blends with the three synthesized plasticizers were adequate compared to commercial biobased plasticizers for PLA. Furthermore, comparison against reported studies on other biobased plasticizers shows that the overall performances of the three synthesized eugenol-based plasticizers were comparable. For example, 10 wt% oregano essential oil that contains phenolic compounds increased the strain at break of PLA to 338%, while the modulus and tensile strength were 1.45 GPa and 29.4 MPa, respectively [45]. With the presence of 20 wt% derivatives of cardanol containing phenolic ester bonds, the tensile stress of PLA declined to 18 MPa by cardanol acetate and to 10 MPa by epoxidized cardanol acetate, meanwhile the tensile strain was increased to 37% by cardanol acetate and to 18% by epoxidized cardanol acetate [48]. In addition, 20 wt% oligoesters enabled the tensile stress and tensile strain of PLA to rise to 30 MPa and 14%, respectively [46].

### 3.5. Antibacterial Properties of The Synthesized Plasticizers

**Figure 6:**
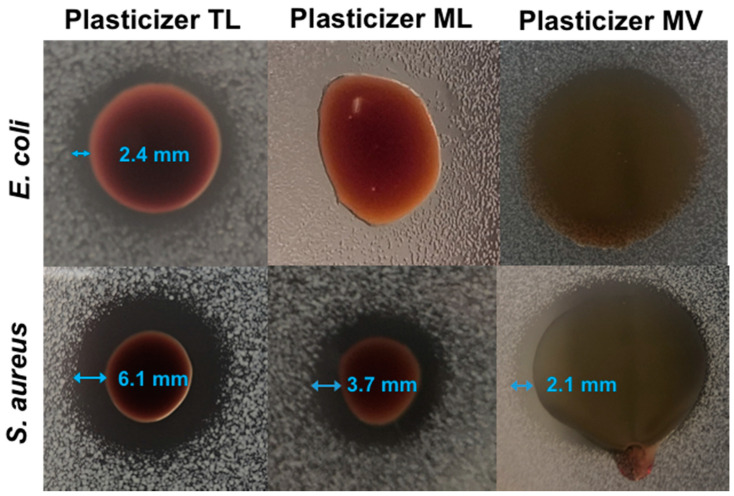
Zone of inhibition test on neat plasticizers against *E. coli* and *S. aureus*.

**Figure 7:**
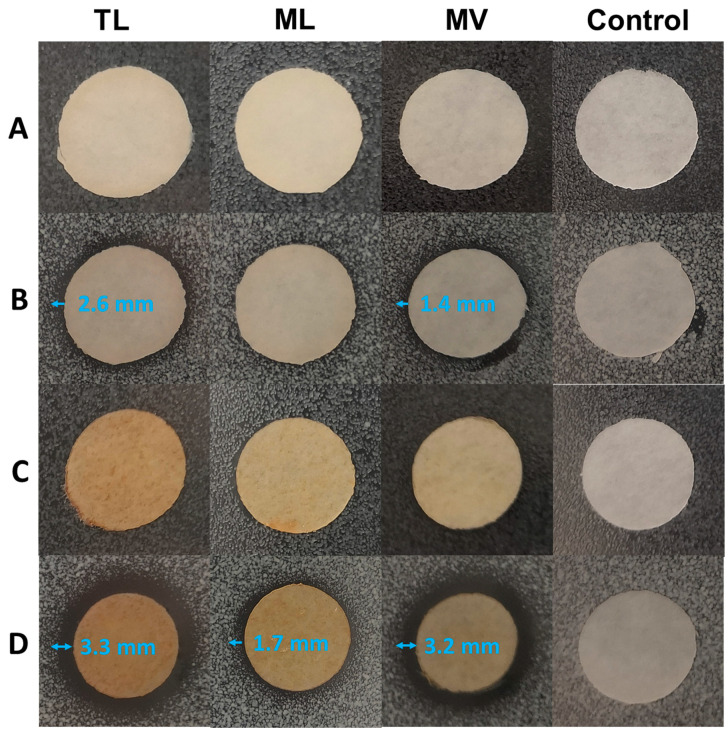
Zone of inhibition test for the plasticizers loaded on paper disks against *E. coli* and *S. aureus* (**A**: 1 mg of plasticizers and *E. coli*; **B**: 1 mg of plasticizers and *S. aureus*; **C**: 5 mg of plasticizers and *E. coli*; **D**: 5 mg of plasticizers and *S. aureus*).

The bactericidal activity of the plasticizers TL, ML and MV were assessed by a zone of inhibition test against Gram-positive *Staphylococcus aureus* (*S. aureus*) and Gram-negative *Escherichia coli* (*E. coli*) in three different forms—PLA disks plasticized by 10 and 20 wt% plasticizer, paper disks loaded with 1 or 5 mg plasticizers and spots with neat plasticizers. The results indicated that the plasticizer TL exhibited stronger bactericidal activity for both *E. coli* and *S. aureus*, as compared to plasticizer ML and MV that demonstrated bactericidal activity only for *S. aureus* (Figure 6 and Figure 7). The bactericidal activity on an agar plate was affected by the diffusion conditions of plasticizers on the agar plate (migration rate of plasticizer from PLA blend and water solubility of the plasticizer), the concentration of plasticizer and the intrinsic antibacterial capability of the plasticizer. As seen in Figure 6, part of the plasticizer droplets did not level and spread uniformly on the surface of agar plates due to the high viscosity of the plasticizer. The diffusion ability of each plasticizer also varied due to the chemical structure. The weight, volume and shape of each droplet were not the same. As a consequence, only qualitative bactericidal activity of the plasticizer was obtained. The gap distances between the droplets and inhibition zones were presented in Figure 6 and Table S11. The migration behavior of a plasticizer is influenced by its structure [49,50,51,52]*,* and, due to the limited migration of the plasticizers from PLA blends, no clear inhibition zone was observed in Figure S36. Therefore, the bactericidal activity of the plasticizer was further evaluated by testing paper disks loaded with 1 or 5 mg of plasticizers. As expected, a higher concentration of antibacterial plasticizer resulted in a larger inhibition zone on the agar plate (Figure 7 and Table S12). Generally, phenolic hydroxyl groups and alcohol groups are both considered as active functionalities for antibacterial effects [53,54]. The former was widely present in all three plasticizers and the latter existed in TL with a significantly higher concentration as compared to plasticizers ML and MV, according to Table S2. In agreement, plasticizer TL, with the highest content of phenolic hydroxyl groups and alcohol groups, had the highest bactericidal activity among the three plasticizers. Moreover, plasticizer TL also had the highest content of carboxylic groups, which might lead to improved water solubility and strengthen the bactericidal activity due to the dissociation of protons.

## 4. Conclusions

Three eugenol-based plasticizers were successfully prepared by a one-pot reaction between eugenol and the green platform chemical levulinic acid, or eugenol and valeric acid. The complicated structures of the synthesized plasticizes were defined by a library of techniques (^1^ H NMR, ^13^ C NMR, ^31^ P NMR, HSQC, COSY, HMBC and ESI-MS). The relationship between polar and nonpolar groups in the chemical structure and the molecular weight of the synthesized plasticizers governed the plasticization efficiency and the mechanical properties of the resulting PLA blends. The *T*_g_ of all plasticized PLA blends decreased considerably compared to neat PLA. All the synthesized plasticizers also increased the strain at break of PLA. The MV (eugenyl valerate) plasticizer enabled the largest depression in *T*_g_ and largest strain at break compared to other PLA blends with same plasticizer concentration. This is put down to lower molecular weight, the highest concentration of ester groups for forming secondary interactions with PLA as well as a longer flexible alkane chain, assumed to lead to higher plasticization effect. The antibacterial assessment proved the bactericidal activity of the three plasticizers against *Staphylococcus aureus*. The plasticizer eugenyl levulinate also inhibited the growth of *Escherichia coli* in zone of inhibition tests. Hence, the plasticizers derived from eugenol and levulinic acid demonstrated favorable dual functions to act as antibacterial plasticizers for PLA.

## Supplementary Materials

The following are available online at https://www.mdpi.com/2218-273X/10/7/1077/s1, Figures S1–S22 and Tables S1–S6: The structural analyses of plasticizer MV, ML and TL; Figure S23 and Tables S7–S9: Thermal stability of plasticizers and their PLA blends; Figures S24–S35 and Table S10: Mechanical performance of plasticized PLA; Figure S36 and Tables S11, S12: Antibacterial study of the plasticizers.
