# Supplementary material for: Dual-Functioning Antibacterial Eugenol-Derived Plasticizers for Polylactide
Source: Biomolecules. 2020 Jul 20;10(7):1077. doi: 10.3390/biom10071077 (PMC7407572; doi:10.3390/biom10071077)
Supplement: Supplementary file 1 [file biomolecules-10-01077-s001.pdf]

Supporting Information

Dual-functioning Antibacterial Eugenol-derived Plasticizer for Polylactide

Wenxiang Xuan [1], Karin Odelius [1] and Minna Hakkarainen [1,*]

[1] Department of Fibre and Polymer Technology, School of Engineering Sciences in Chemistry, Biotechnology and Health, KTH Royal Institute of Technology, Teknikringen 56, Stockholm, 100 44, Sweden

* Correspondence: minna@kth.se

Number of pages: 30

Number of figures: 36

Number of tables: 12

The structural analyses of plasticizer MV, ML and TL.

In total four reactions were performed, including a control reaction with only eugenol, and the obtained products were characterized by a series of techniques. Three of the obtained products (TL, ML and MV) were further evaluated as antibacterial plasticizers for PLA. The details of reactions and products were summarized in the table below.

	**Reagents**	**Catalyst**	**Temp.**	**Reaction Time**	**Abbreviation for obtained plasticizer**
**Control Reaction**	Eugenol	*p*-toluene sulfonic acid monohydrate, 1 mol% equiv. to eugenol	140 °C	24 hours	/
**Synthesis 1**	Eugenol:Levulinic acid = 3:1 in mole				TL
**Synthesis 2**	Eugenol:Levulinic acid = 1:1 in mole				ML
**Synthesis 3**	Eugenol:Valeric acid = 1:1 in mole				MV

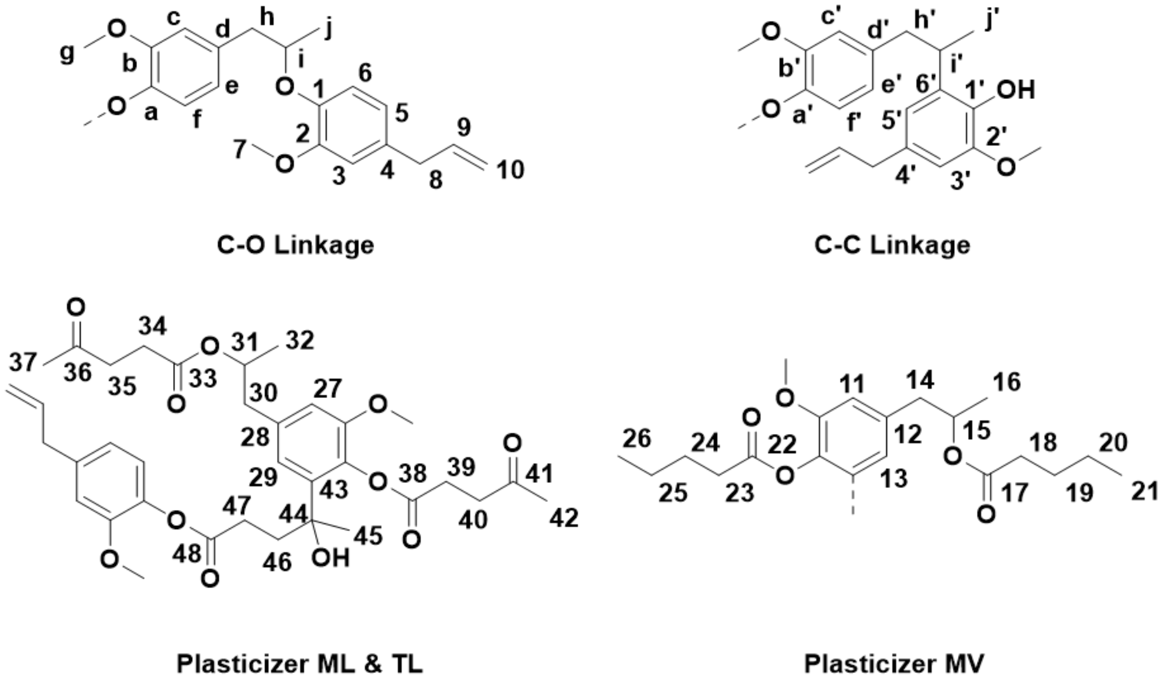

Figure S1. Atom numbering for correlation signal assignments in 2D NMR.

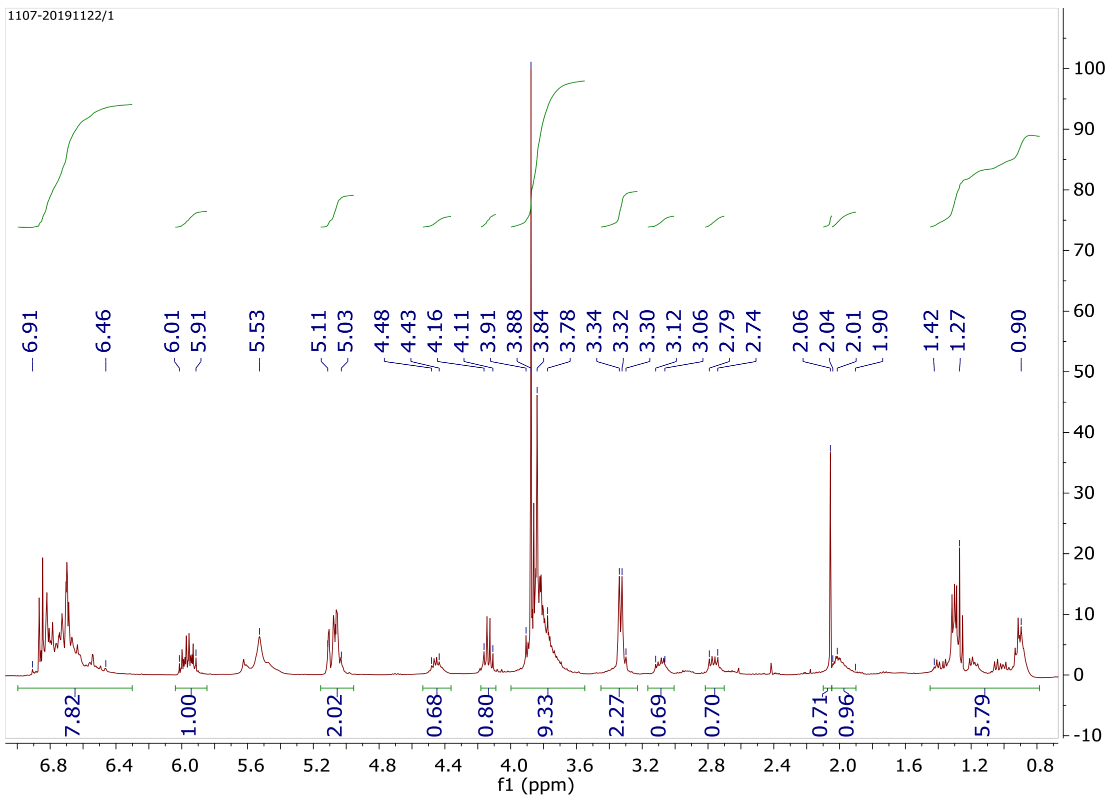

Figure S2. ^{1}H NMR spectrum of the product from control reaction with only eugenol.

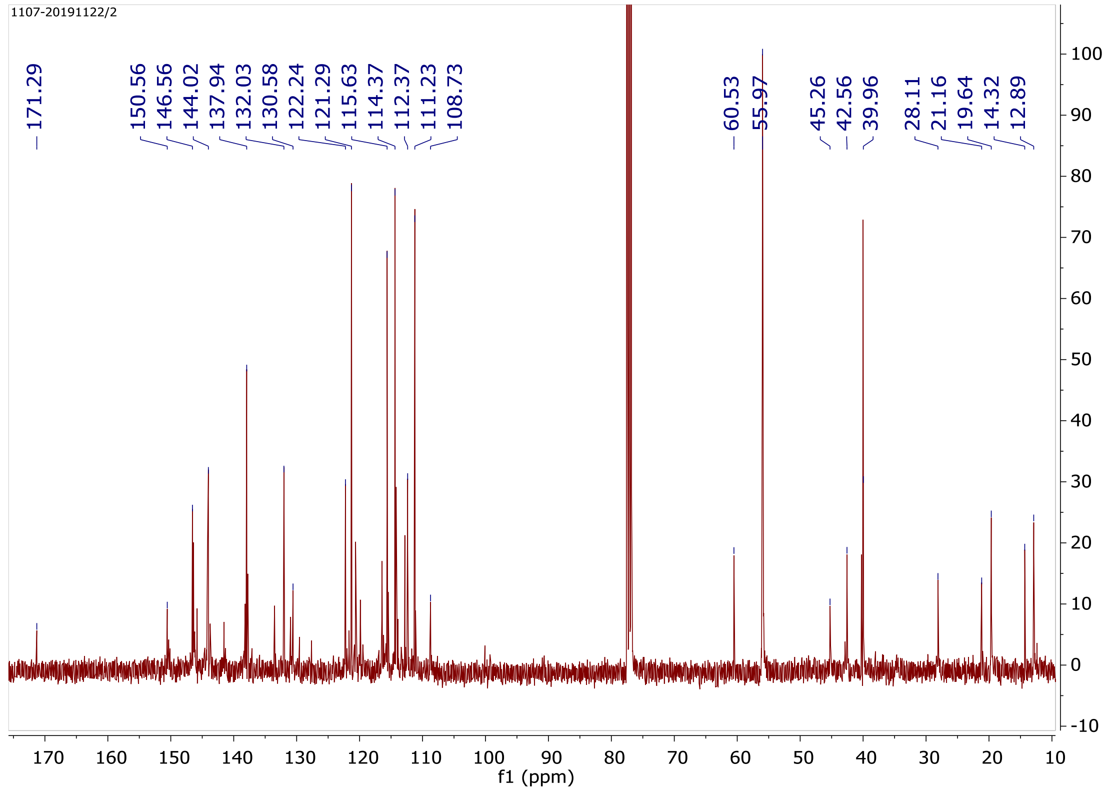

Figure S3. ^{13}C NMR spectrum of the product from control reaction with only eugenol (the

carbonyl carbon signal generated from solvent ethyl acetate).

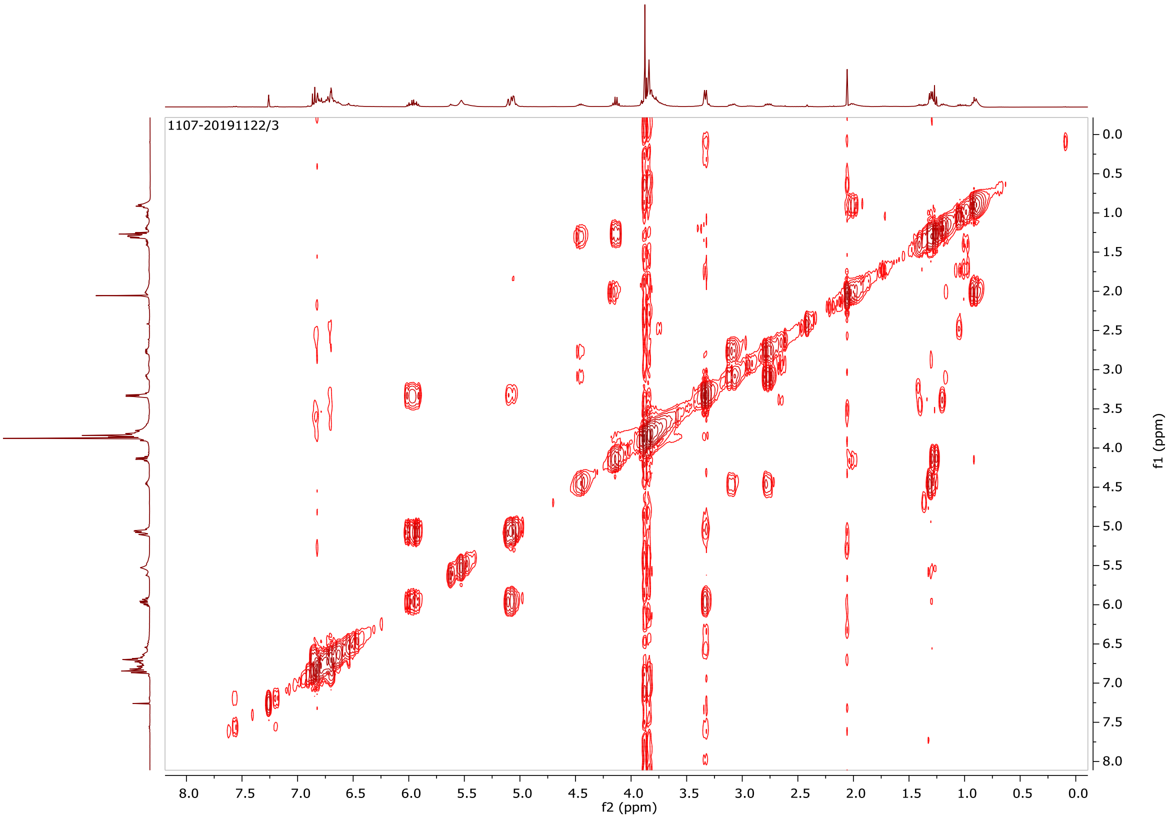

Figure S4. COSY spectrum of the product from control reaction with only eugenol.

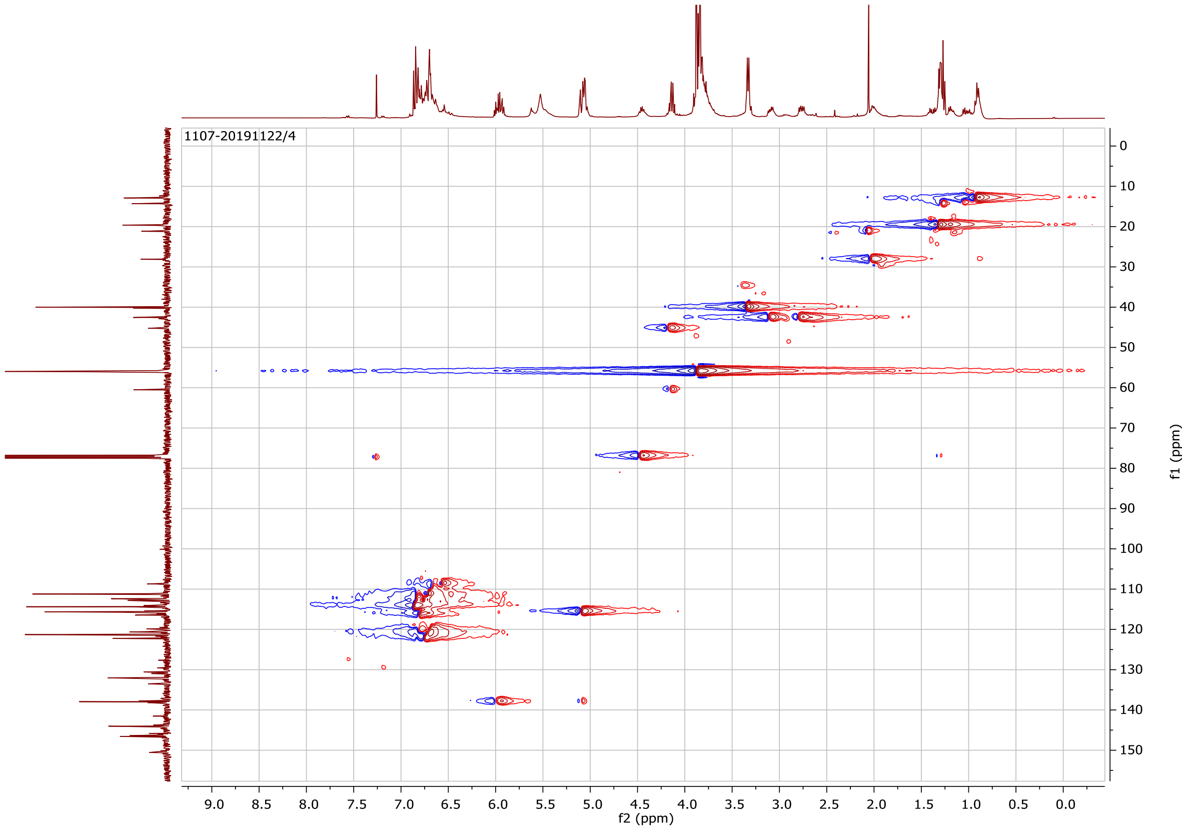

Figure S5. HSQC spectrum of the product from control reaction with only eugenol.

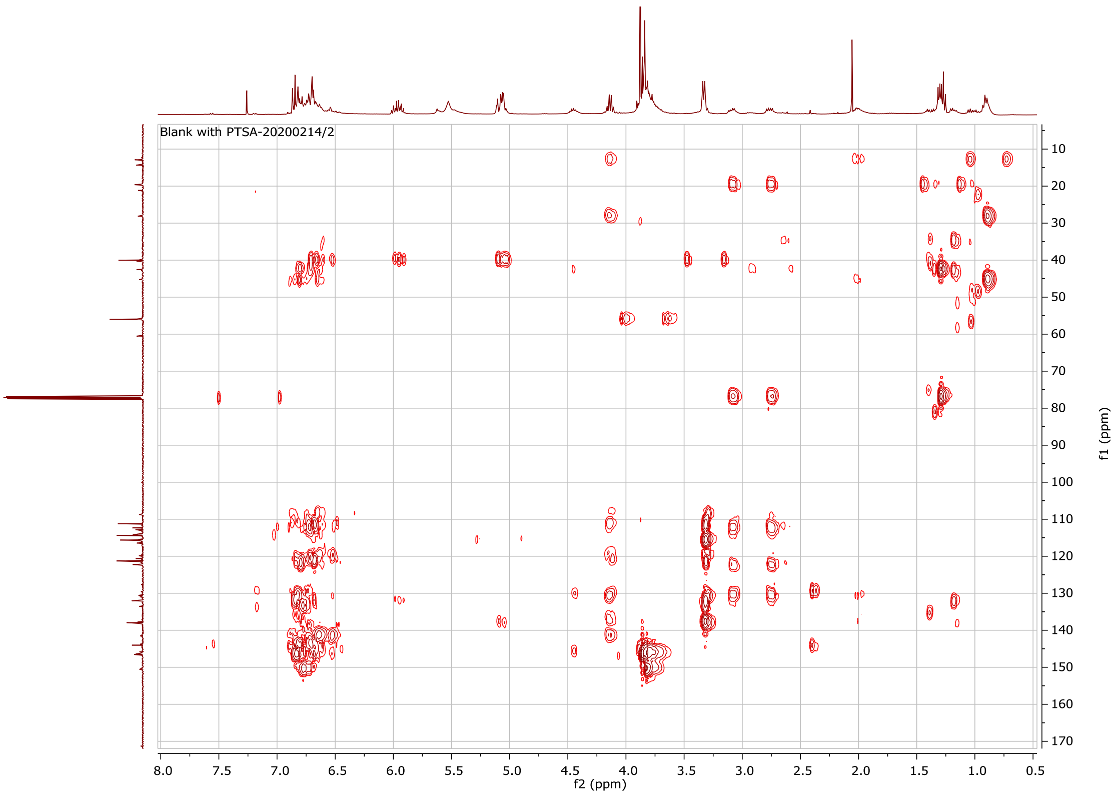

Figure S6. HMBC spectrum of the product from control reaction with only eugenol (calibrated by δ_H/δ_C = 1.29/76.80 ppm).

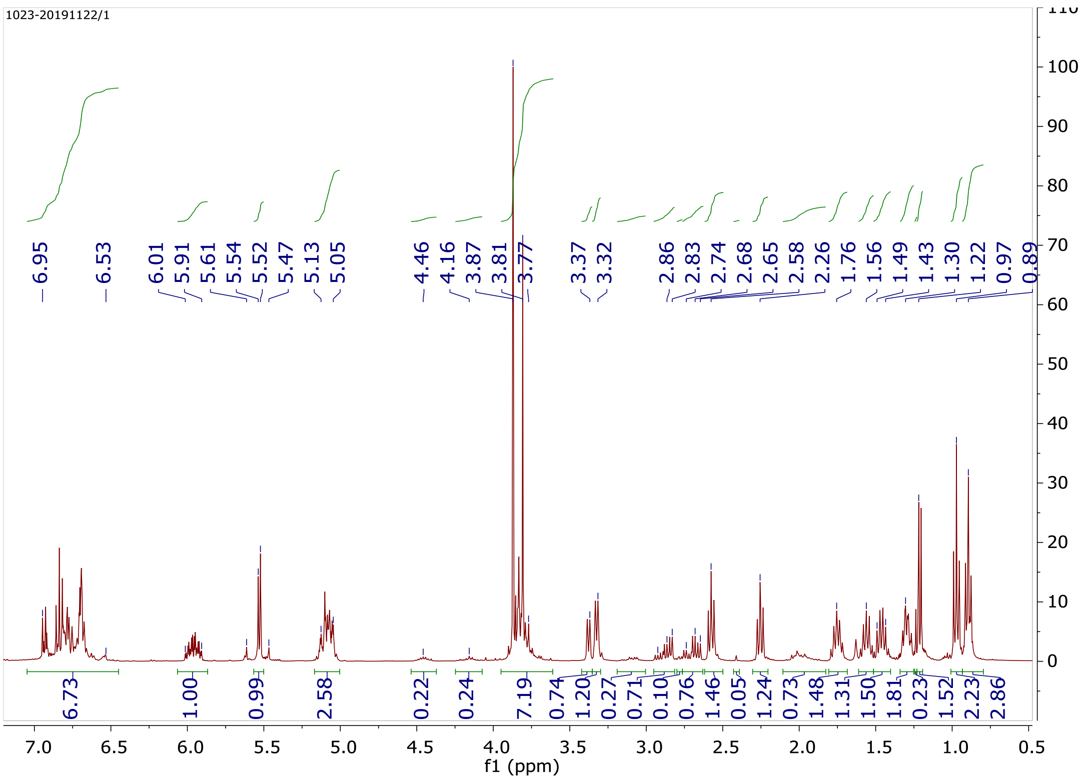

Figure S7. ^{1}H NMR spectrum of plasticizer MV.

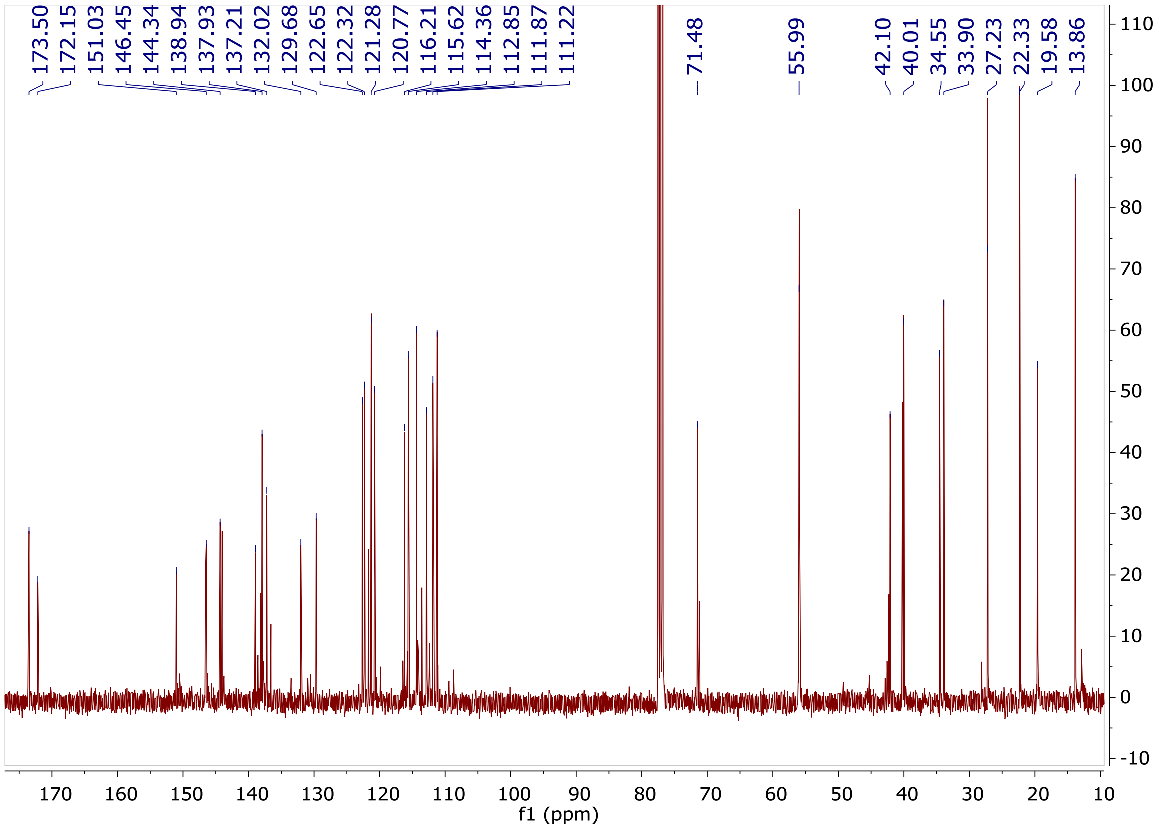

Figure S8. ^{13}C NMR spectrum of plasticizer MV.

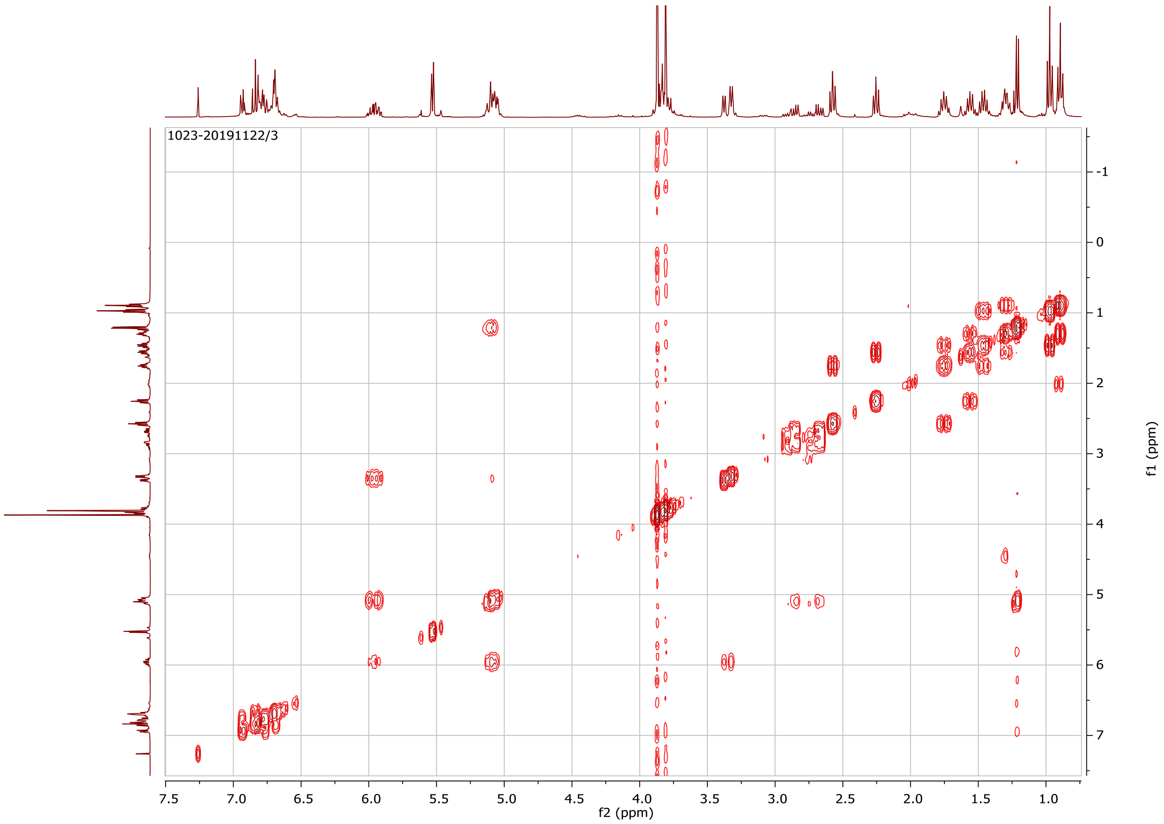

Figure S9. COSY spectrum of plasticizer MV.

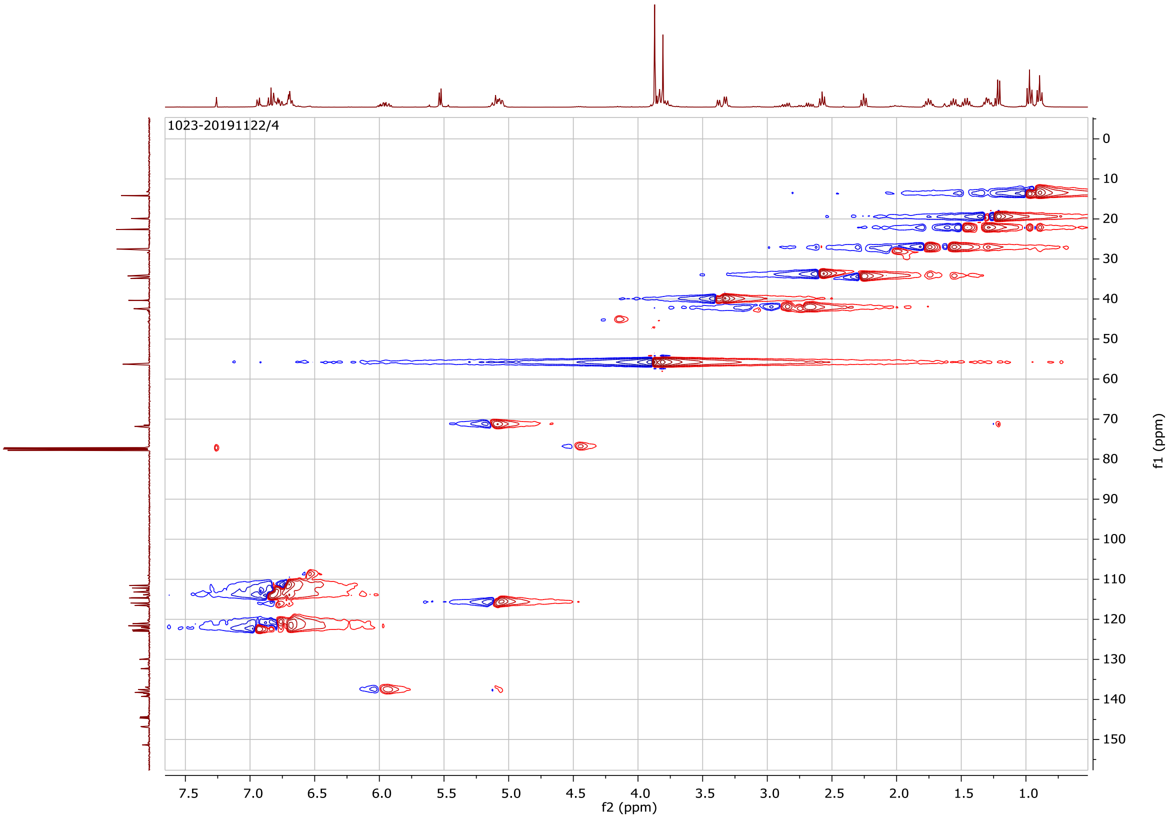

Figure S10. HSQC spectrum of plasticizer MV.

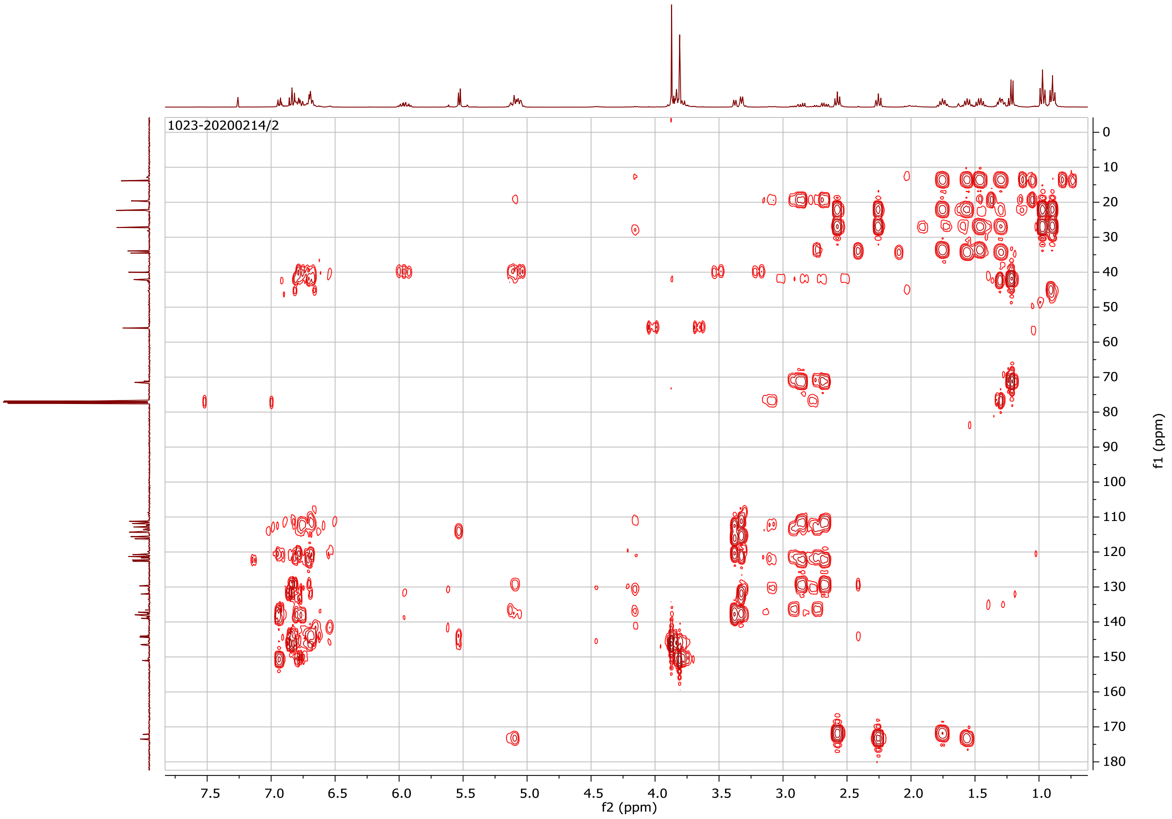

Figure S11. HMBC spectrum of plasticizer MV (calibrated by δ_H/δ_C = 1.21/71.24 ppm).

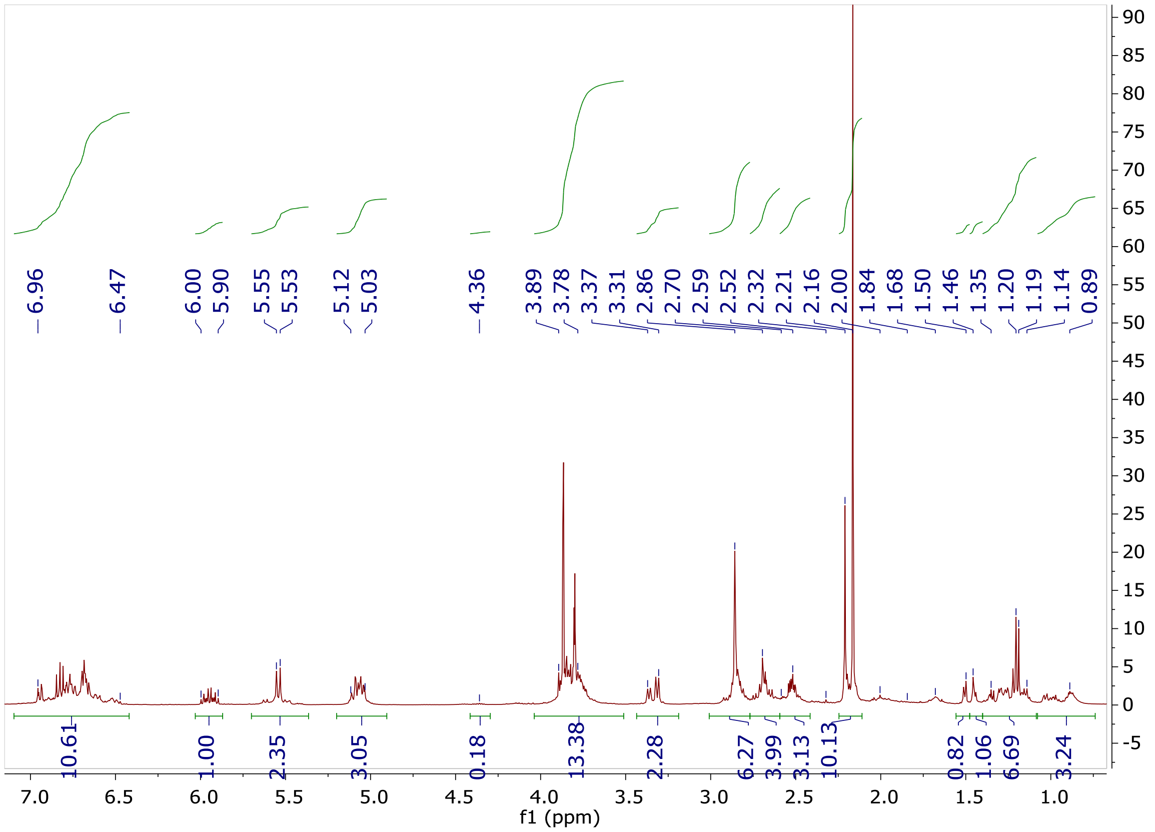

Figure S12. ^{1}H NMR spectrum of plasticizer ML.

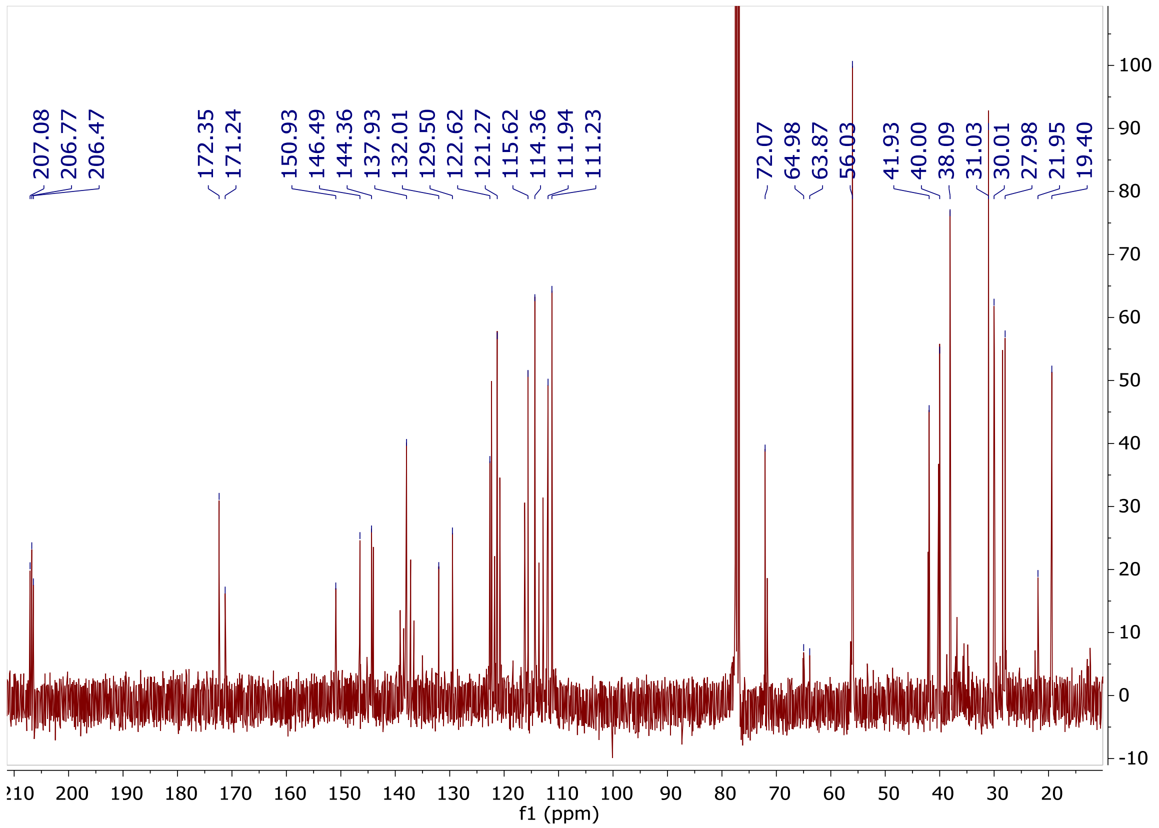

Figure S13. ^{13}C NMR spectrum of plasticizer ML.

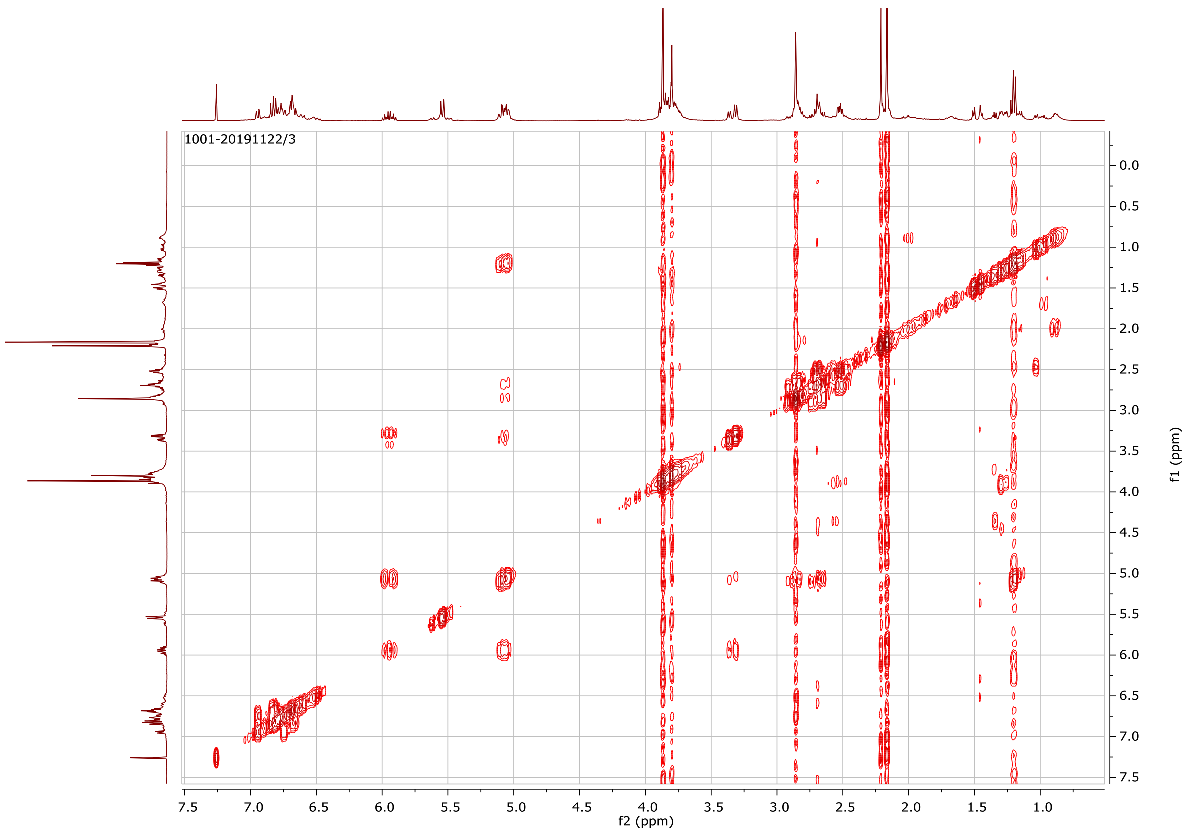

Figure S14. COSY spectrum of plasticizer ML.

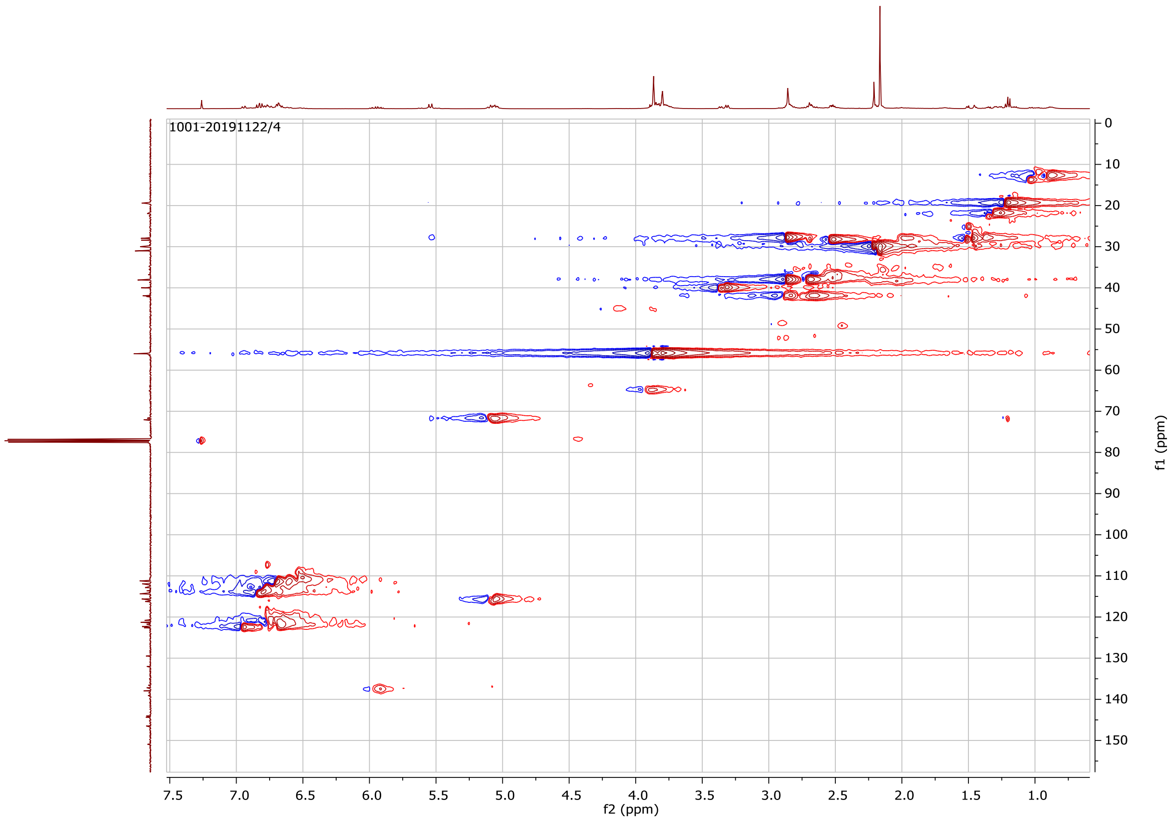

Figure S15. HSQC spectrum of plasticizer ML.

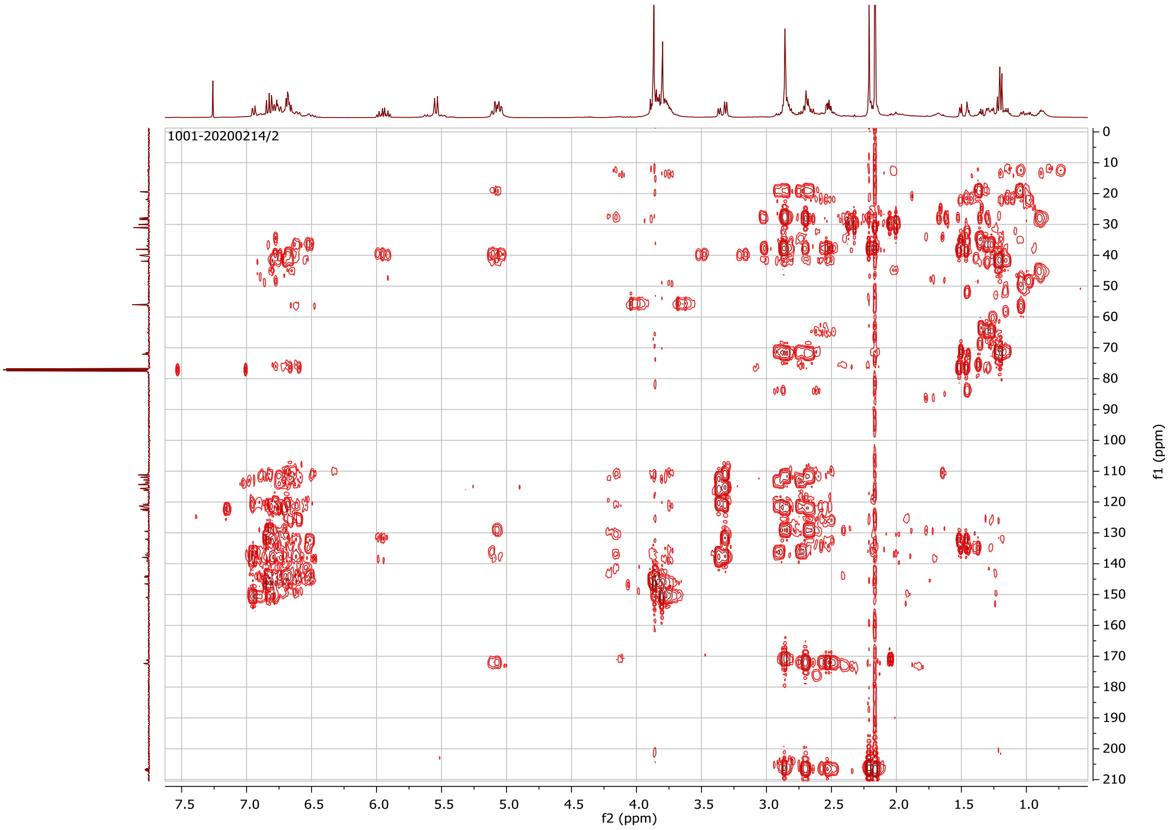

Figure S16. HMBC spectrum of plasticizer ML (calibrated by δ_H/δ_C = 1.20/71.77 ppm).

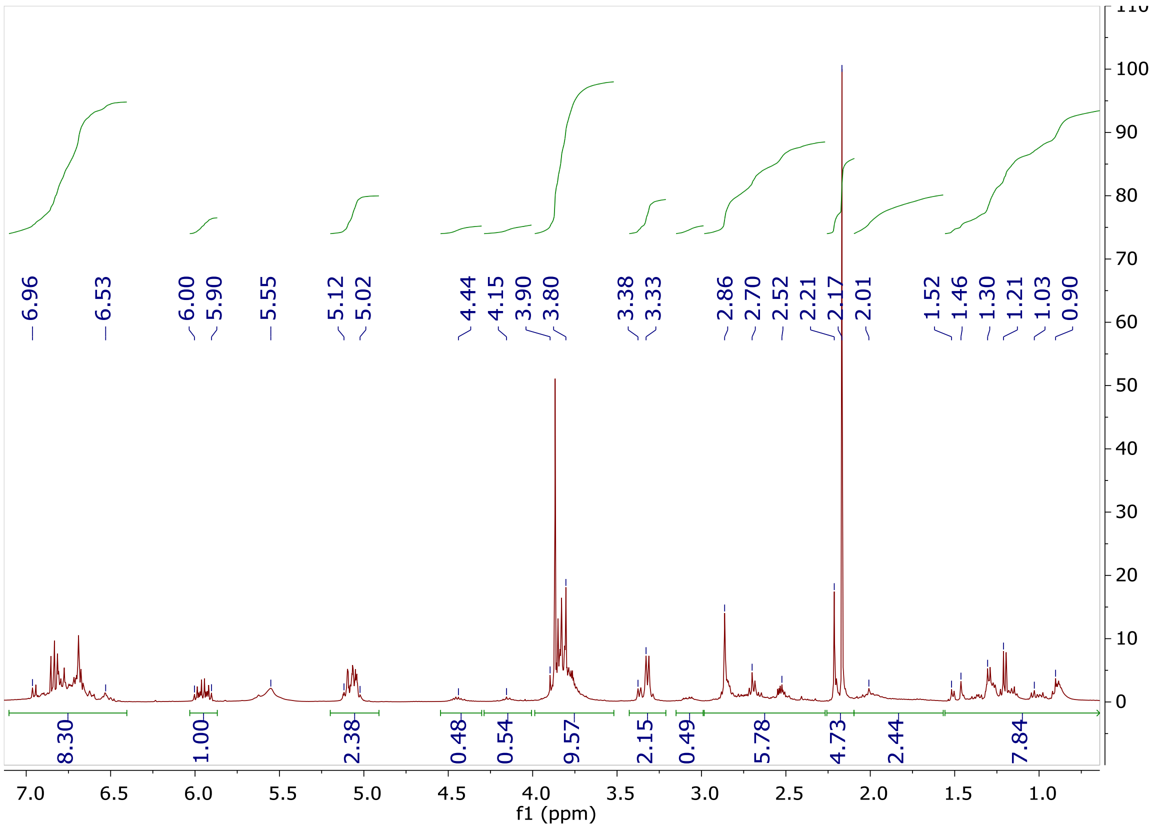

Figure S17. ^{1}H NMR spectrum of plasticizer TL.

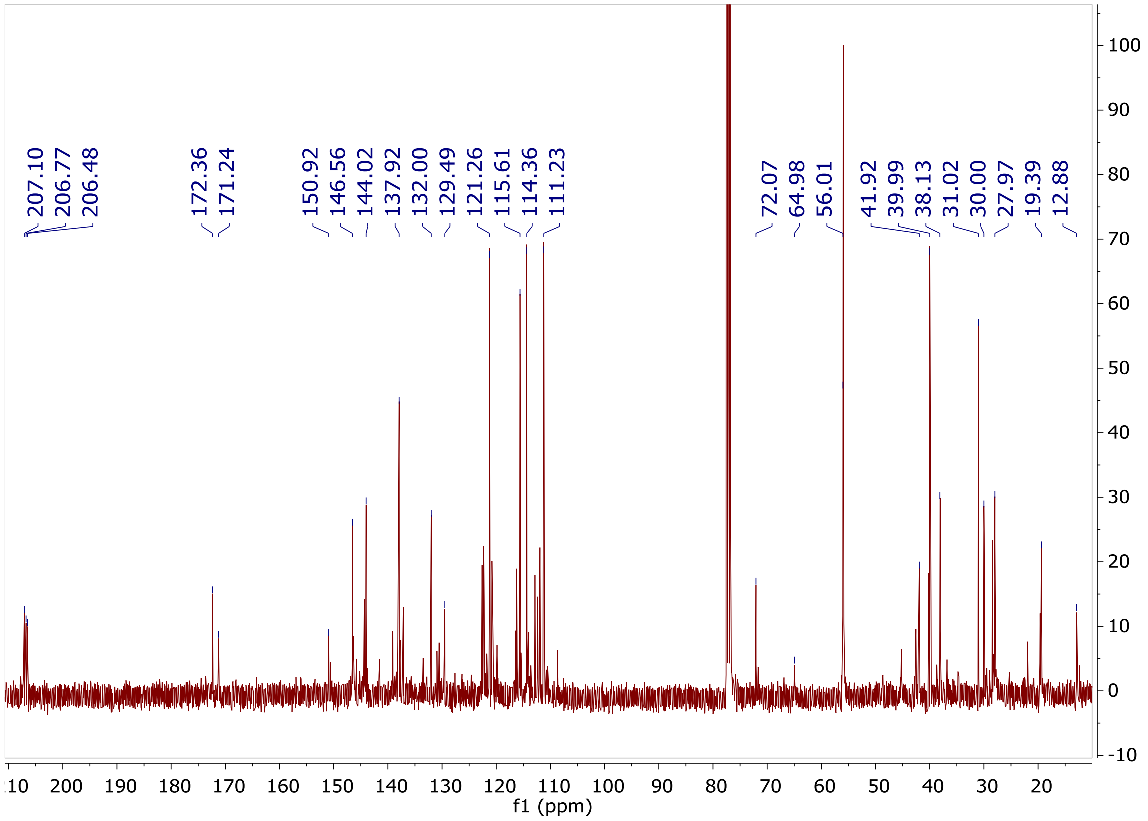

Figure S18. ^{13}C NMR spectrum of plasticizer TL.

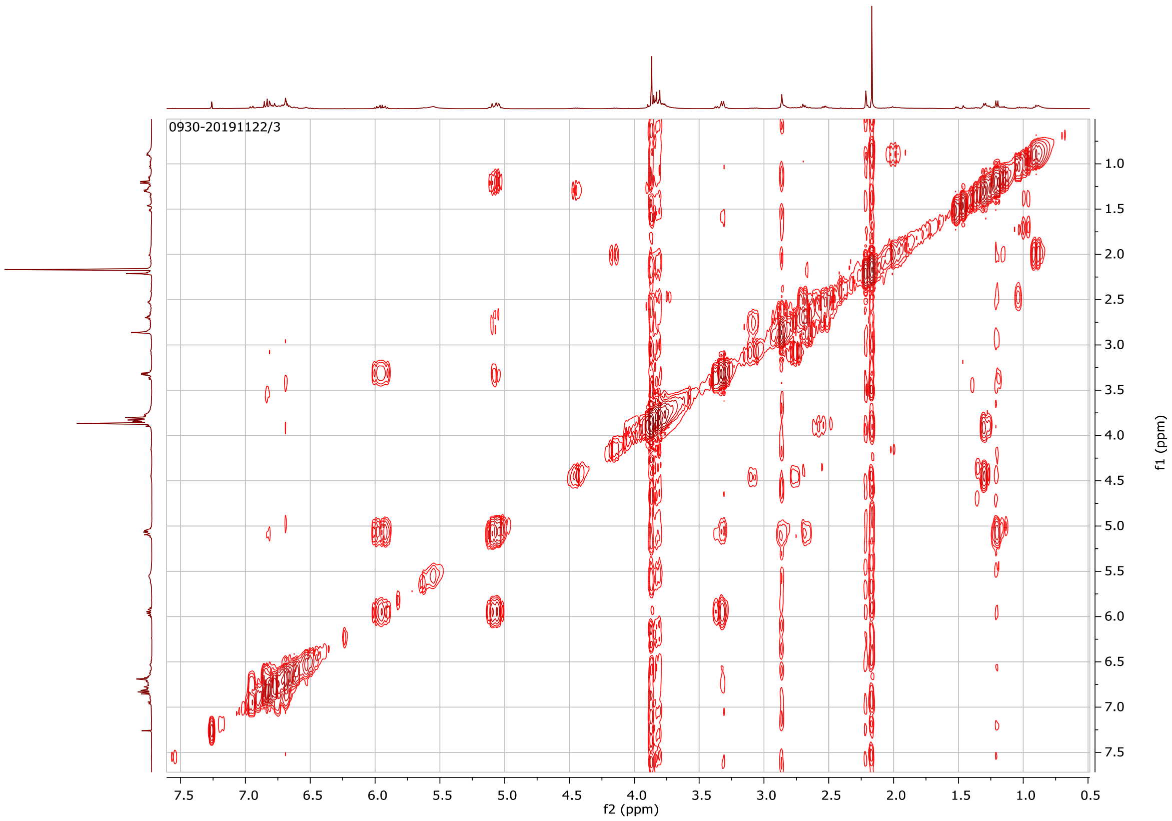

Figure S19. COSY spectrum of plasticizer TL.

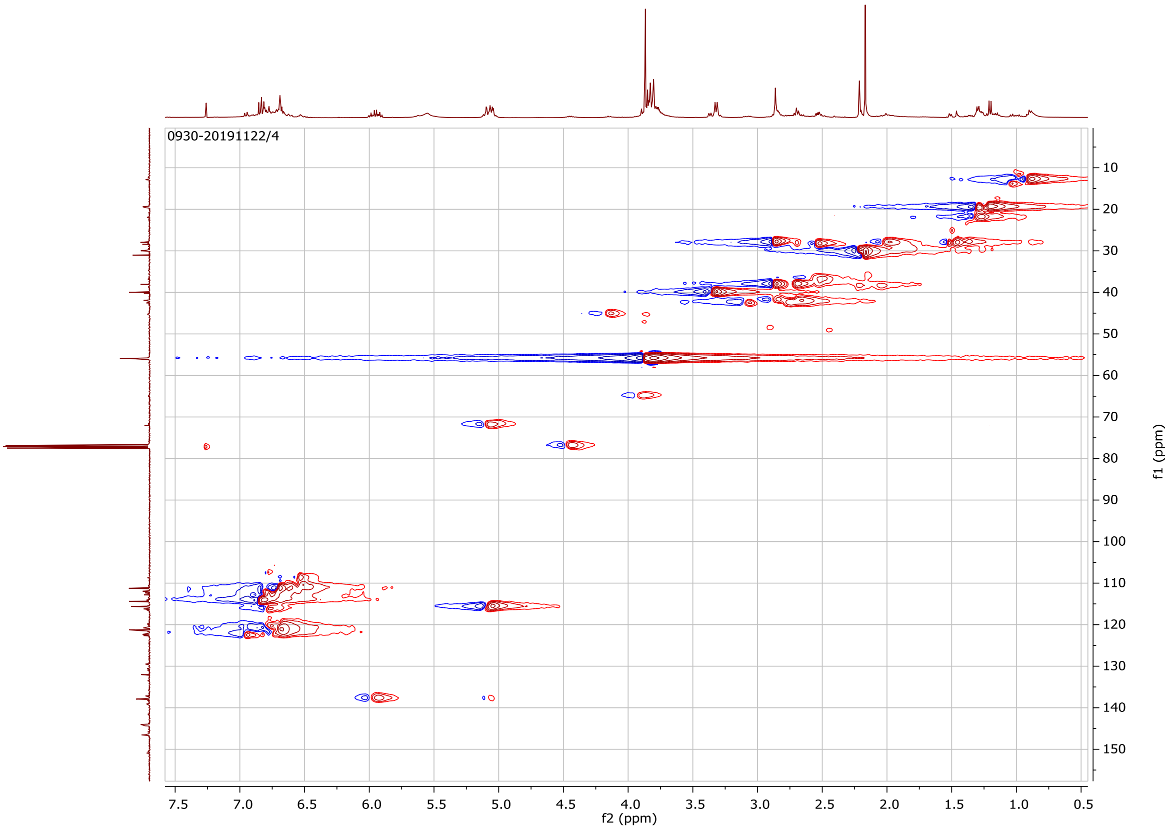

Figure S20. HSQC spectrum of plasticizer TL.

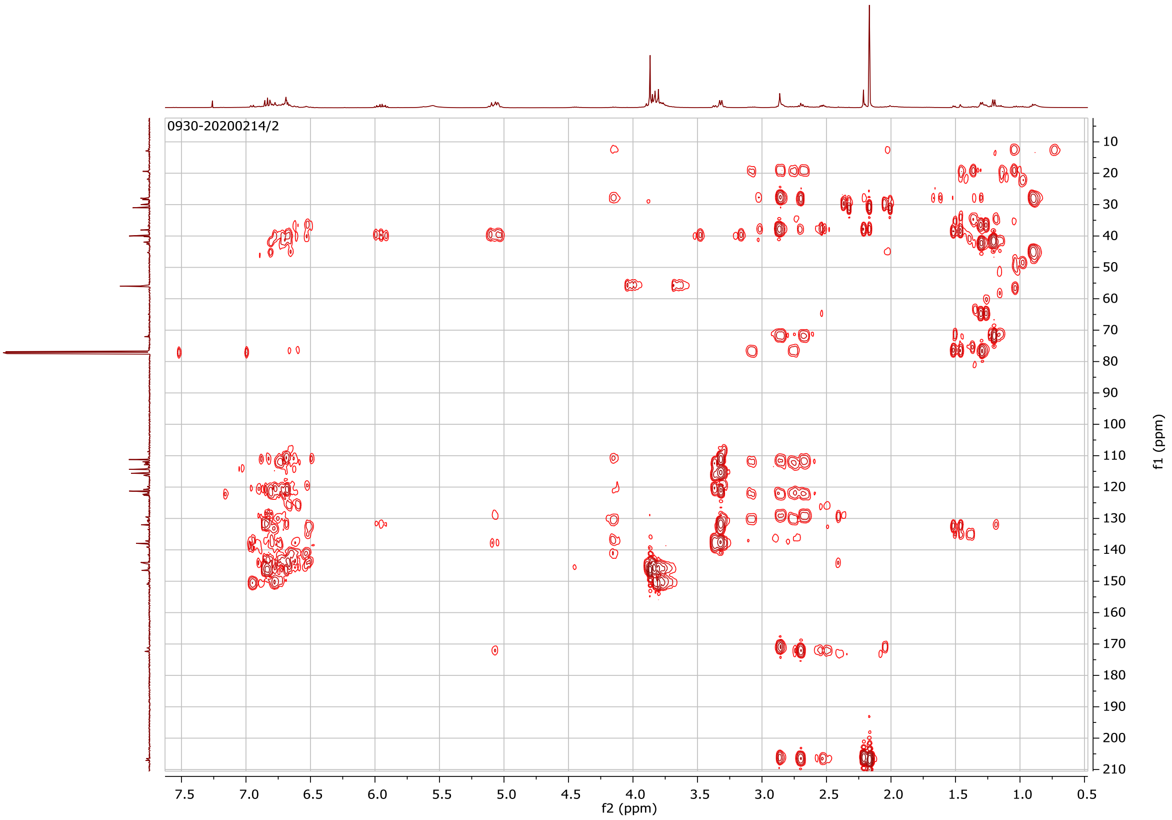

Figure S21. HMBC data of plasticizer TL (calibrated by δ_H/δ_C = 1.20/71.79 ppm).

Table S1. Ratios of selected proton integrations from ^{1}H NMR analyses of four obtained reaction products.

Product	Feeding ratio of starting materials	Arene/ Methoxyl	Alkene/ Methoxyl	Methyl (< 1.5 ppm)/Methoxyl	Methyl (2.1 ppm)/Methoxyl
Product from control reaction with only eugenol	Eugenol only	7.82/9.33 = 0.84	3.00/9.33 = 0.32	/	/
MV	Eugenol:Velaric acid = 1:1	6.73/7.19 = 0.94	3.00/7.19 = 0.42	/	/
ML	Eugenol:Levulinic acid = 1:1	10.61/13.38 = 0.79	3.00/13.38 = 0.22	11.81/13.38 = 0.88	10.13/13.38 = 0.76
TL	Eugenol:Levulinic acid = 3:1	8.30/9.57 = 0.87	3.00/9.57 = 0.31	7.49/9.57 = 0.78	4.73/9.57 = 0.49

Table S2. Summary of ^{31}P NMR analyses of neat eugenol and four obtained reaction products.

	Eugenol	Control reaction	TL	ML	MV
Sample Weight (mg)	26.20	31.36	29.68	29.86	30.55
Integration of NHND (equiv. to 3 mg NHND)	1.00	1.00	1.00	1.00	1.00
Integration of Aliphatic OH	0	0	0.14	0.03	0.02
Integration of Phenolic OH (Substituted type)	0	2.26	0.83	0.33	0.55
Integration of Phenolic OH (Non-substituted type)	8.69	6.82	5.27	4.05	4.65
Integration of Carboxylic Acid	0.00	0.00	0.08	0.02	0.01
Overall Phenol Content (mmol/g)	5.55	4.85	3.44	2.46	2.85
Alcohol Content (mmol/g)	0.00	0.00	0.08	0.02	0.01
Carboxyl Content (mmol/g)	0.00	0.00	0.05	0.01	0.01
Ratio of Phenolic OH (Substituted:Non-substituted)	0.00	0.33	0.16	0.08	0.12

Table S3. ESI-MS peak assignment for the product from control reaction with only eugenol.

**m/z**	**Corresponding Components**
352	2 Eugenol + Na^+
515	3 Eugenol + Na^+
680	4 Eugenol + Na^+
845	5 Eugenol + Na^+
1009	6 Eugenol + Na^+
1173	7 Eugenol + Na^+

Table S4. ESI-MS peak assignment of plasticizer MV.

m/z	Corresponding Components	Involved Reactions
271	1 Eugenol + 1 VaA - H_2O + Na^+	1 dehydration
289	1 Eugenol + 1 VaA + Na^+	1 addition
373	1 Eugenol + 2 VaA - H_2O + Na^+	1 addition & 1 dehydration
435	2 Eugenol + 1 VaA - H_2O + Na^+	1 dehydration
520	2 Eugenol + 2 VaA - 2 H_2O + Na^+	2 dehydrations
537	2 Eugenol + 2 VaA - 1 H_2O + Na^+	1 addition & 1 dehydration
600	3 Eugenol + 1 VaA - 1 H_2O + Na^+	1 dehydration
622	2 Eugenol + 3 VaA - 2 H_2O + Na^+	1 addition & 2 dehydrations
684	3 Eugenol + 2 VaA - 2 H_2O + Na^+	2 dehydrations
702	3 Eugenol + 2 VaA - 1 H_2O + Na^+	1 addition & 1 dehydration
768	3 Eugenol + 3 VaA - 3 H_2O + Na^+	3 dehydrations
786	3 Eugenol + 3 VaA - 2 H_2O + Na^+	1 addition & 2 dehydrations
848	4 Eugenol + 2 VaA - 2 H_2O + Na^+	2 dehydrations

Table S5. ESI-MS peak assignment of plasticizer ML.

m/z	Corresponding Components	Involved Reactions
401	1 Eugenol + 2 LeA - H_2O + Na^+	1 addition & 1 dehydration
449	2 Eugenol + 1 LeA - H_2O + Na^+	1 dehydration
547	2 Eugenol + 2 LeA - 2 H_2O + Na^+	2 dehydrations
565	2 Eugenol + 2 LeA - 1 H_2O + Na^+	1 addition & 1 dehydration
664	2 Eugenol + 3 LeA - 2 H_2O + Na^+	1 addition & 2 dehydrations
828	3 Eugenol + 3 LeA - 2 H_2O + Na^+	1 addition & 2 dehydrations
927	3 Eugenol + 4 LeA - 3 H_2O + Na^+	1 addition & 3 dehydrations

Table S6. ESI-MS peak assignment of plasticizer TL.

m/z	Corresponding Components	Involved Reactions
285	1 Eugenol + 1 LeA - H_2O + Na^+	1 dehydration
401	1 Eugenol + 2 LeA - H_2O + Na^+	1 addition & 1 dehydration
449	2 Eugenol + 1 LeA - H_2O + Na^+	1 dehydration
467	2 Eugenol + 2 LeA + Na^+	2 additions
547	2 Eugenol + 2 LeA - 2 H_2O + Na^+	2 dehydrations
565	2 Eugenol + 2 LeA - 1 H_2O + Na^+	1 addition & 1 dehydration
614	3 Eugenol + 1 LeA - 1 H_2O + Na^+	1 dehydration
664	2 Eugenol + 3 LeA - 2 H_2O + Na^+	1 addition & 2 dehydrations
712	3 Eugenol + 2 LeA - 2 H_2O + Na^+	2 dehydrations
730	3 Eugenol + 2 LeA - 1 H_2O + Na^+	1 addition & 1 dehydration
778	4 Eugenol + 1 LeA - 1 H_2O + Na^+	1 dehydration
828	3 Eugenol + 3 LeA - 2 H_2O + Na^+	1 addition & 2 dehydrations
877	4 Eugenol + 2 LeA - 2 H_2O + Na^+	2 dehydrations

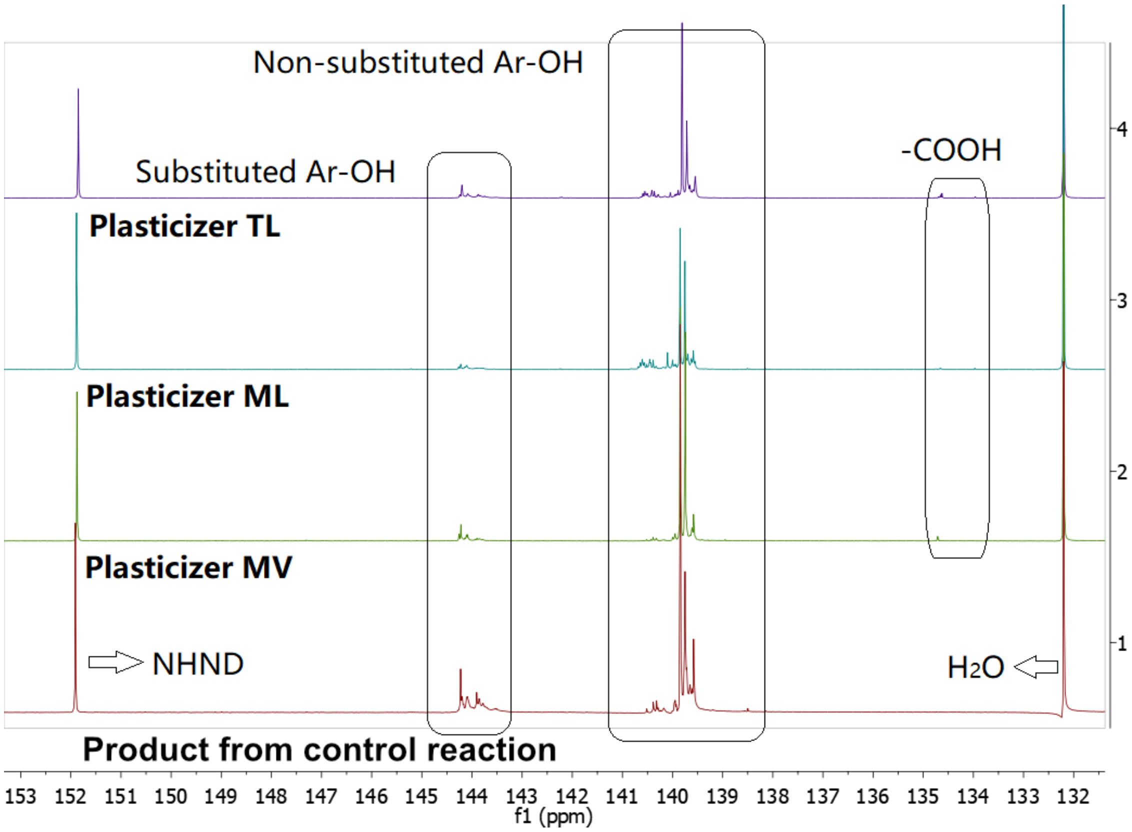

Figure S22. ^{31}P NMR spectra of the product from control reaction with only eugenol, plasticizer MV, ML and TL.

Three plasticizer candidates, plus the products from control reaction with only eugenol, were examined by ^{1}H NMR, ^{13}C NMR and ^{31}P NMR to characterize the functionalities, and together with COSY, HSQC and HMBC to define the structural linkages. With the help of combined NMR and ESI-MS analyses, probable molecular structures were deduced.

The products from control reaction with only eugenol. consisted of a series of single sodium ion adducts of eugenol oligomers, as shown in ESI-MS, **Figure 2a** and **Table S3**. The results from ^{31}P NMR, **Figure S22** and **Table S2**, showed the oligomers had a lower phenolic hydroxyl group content (4.85 mmol/g) than the starting materials (6.09 mmol/g, theoretical value). The alkene and arene protons were also consumed in the reaction, as indicated by the decreasing ratio of integration of alkene (or arene) towards methoxyl groups in ^{1}H NMR, **Figure S2** and **Table S1**. The methoxyl group was considered relatively stable in this case since harsh conditions of demethylation were not utilized. Therefore, it was likely that the phenolic hydroxyl groups, alkene and arene protons were involved in the reaction. The COSY, **Figure S4**, characterized coupling signals from isopropyl chains, **Figure S1**, determined Ci was the reaction site which followed the Markovnikov's Rule. Mainly two types of linkage formed the oligomers, i-O-1 bonds and i'-6' bonds, both were confirmed by HMBC, **Figure S6**. As shown in **Figure S1** and **S6**, the correlation signals between C1 (145.59 ppm) and Hi (4,45 ppm) and correlation signals among Hi' (4.13 ppm), C5' (120.72 ppm), C1' (141.33 ppm), Cj' (12.65 ppm), C6' (137.06 ppm) and Cd' (130.57 ppm) indicated the presence of i-O-1 bonds and i'-6' bonds respectively. The presence of substituted phenolic hydroxyl groups (δ_P = 143.53 - 144.23 ppm),

detected in ^{31}P NMR spectrum, **Figure S22**, further suggested the formation of i′-6′ bonds. The ratio of amount of i-O-1 bond and i′-6′ bond was 52:16 = 3.25 if no side reactions were considered.

Compared to the control reaction, the addition of valeric acids led to a group of eugenyl valerates (plasticizer candidate MV), as shown in ESI-MS, **Figure 2b** and **Table S4**. Similarly, the joint results from ^{1}H NMR, **Figure S7**, HMBC, **Figure S11**, and ^{31}P NMR, **Table S2**, indicated the exist of i-O-1 bonds and i′-6′ bonds. However, the formation of those bonds was significantly inhibited by the competitive reactions of carboxylic acids, addition of carboxyl on alkene double bond and dehydration on phenolic hydroxyl group. Ester bonds were consequently produced which were reflected by two chemical shift peaks (δ_C =172.15, 173.50 ppm) in ^{13}C NMR spectrum, **Figure S8**. Those ester linkages were further validated by HMBC, **Figure S11.** The proton H23 coupling with C22, C24 and C25 symbolized the existence of phenolic ester bonds. The correlation of protons $H14_a$ and $H14_b$ with C11, C12, C13, C15 and C16, and proton H15 with C12 and C17, demonstrated the presence of aliphatic ester bonds created by the direct addition of carboxyl on alkene double bonds.

Levulinic acid had a similar structure to valeric acid and the behaviors of its carboxyl groups in reactions could be predicted based on the previous analyses on the reaction of eugenol and valeric acid. But its extra ketone functionality played a special role in the reactions with eugenol. The ESI-MS spectra, **Figure 2c** and **2d**, suggested two families of distributed products (plasticizer candidate ML and TL) and their peak assignments revealed the existence of additional linkages, **Table S5** and **S6**. Nevertheless, the signals of i-O-1 bonds and i′-6′ bonds that link eugenols disappeared in HMBC, **Figure S16** and **S21**. In addition, based on a comparison of the proton integration, **Table S1**, more methyl groups (0.9 - 1.5 ppm) were obtained than the estimated value (by direct carboxyl addition on alkene) and the methyl groups adjacent to ketone groups (2.1 ppm) and arene protons were both consumed. Hence, nucleophilic addition on ketone groups could be deduced and those linkages were identified by the correlation signals (eg. δ_H/δ_C = 1.46 ppm/76.51, 132.39, 38.39 ppm) in HMBC, **Figure S16**. It has been acknowledged that diphenolic acids can be obtained through condensations of levulinic acids and phenols in the presence of sulfuric acids [1], i.e. the ketone groups can react with arene protons. Moreover, higher alcohol and carboxyl content were found in TL than ML by ^{31}P NMR analysis, **Table S2**, implying that the process of nucleophilic addition on ketone could be influenced by the stoichiometric ratio of reagents. As expected, signals from aliphatic and phenolic ester bonds were clearly recorded in ^{13}C NMR, **Figure S13** and **S18**, and HMBC, **Figure S16** and **S21**. Interestingly, the plasticizer candidate TL had much higher phenolic hydroxyl group content than that of ML, **Table S2**.

Miscibility of PLA with Plasticizer Candidates

Table S7. The glass transition temperatures (T_g) of PLA blends with plasticizer candidates.

Additives / Con. of Additives	TL	ML	MV	Eugenol	LeA
10 wt %	51.5 ± 0.1 °C	51.4 ± 0.3 °C	44.7 ± 0.2 °C	/	/
20 wt %	41.8 ± 0.1 °C	43.2 ± 0.8 °C	29.6 ± 0.3 °C	36.5 ± 5.9 °C	41.4 ± 1.4 °C
30 wt %	36.5 ± 0.3 °C	34.8 ± 0.3 °C	15.6 ± 0.3 °C	/	/

Note: The data was based on the second heating scan of DSC programme. The T_g of PLA100 was 59.4 ± 0.2 °C, from previous work [2].

Thermal Stability of Plasticizer Candidate and its PLA Blends

Table S8. The onset temperatures of 5 % weight loss of PLA blends with plasticizer candidates.

Additives / Con. of Additives	TL	ML	MV	Eugenol	LeA
10 wt %	198 ± 5 °C	207 ± 2 °C	179 ± 1 °C	/	/
20 wt %	174 ± 8 °C	197 ± 2 °C	181 ± 3 °C	139 ± 3 °C	131 ± 1 °C
30 wt %	185 ± 2 °C	195 ± 5 °C	182 ± 3 °C	/	/

Note: The onset temperature of 5 % weight loss for PLA100 was 319 ± 0 °C, from previous work [2].

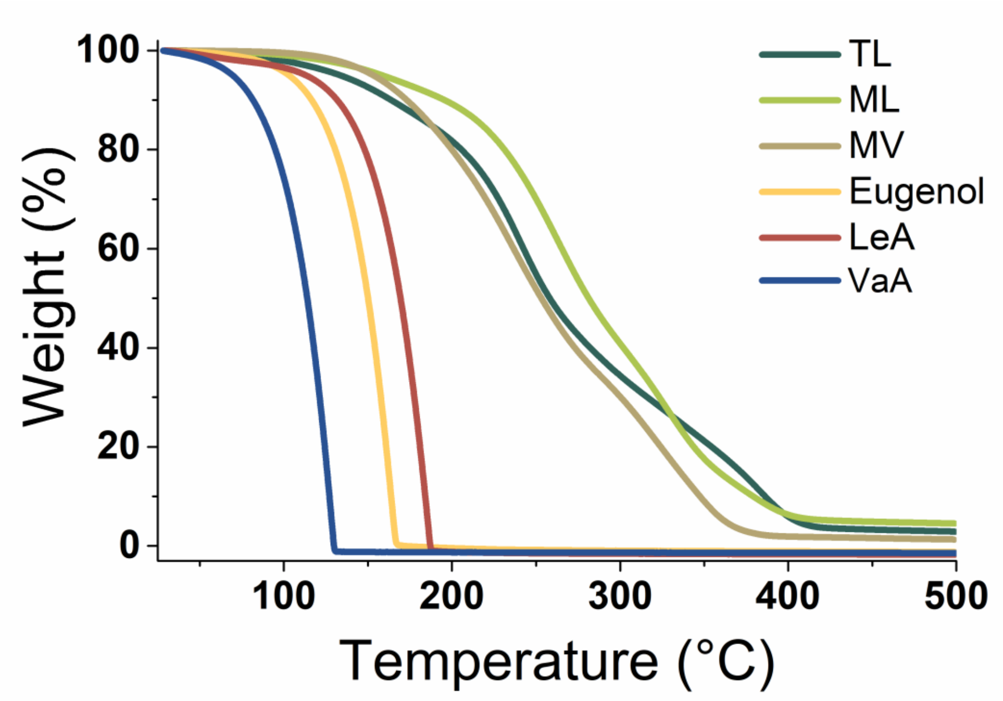

Figure S23. TGA curves of the three plasticizer candidates and their building blocks eugenol, levulinic acid and valeric acid.

Table S9. The onset temperatures of 5 % weight loss (T5) of plasticizer candidates and starting materials (b.p. was stated for starting materials).

	TL	ML	MV	Eugenol	LeA	VaA
T5 (°C)	138.1 ± 3.5	158.1 ± 1.8	143.1 ± 7.3	105.3 ± 2.2	112.9 ± 1.5	73.5 ± 2.9
Boiling point (°C)	/	/	/	254	245	185

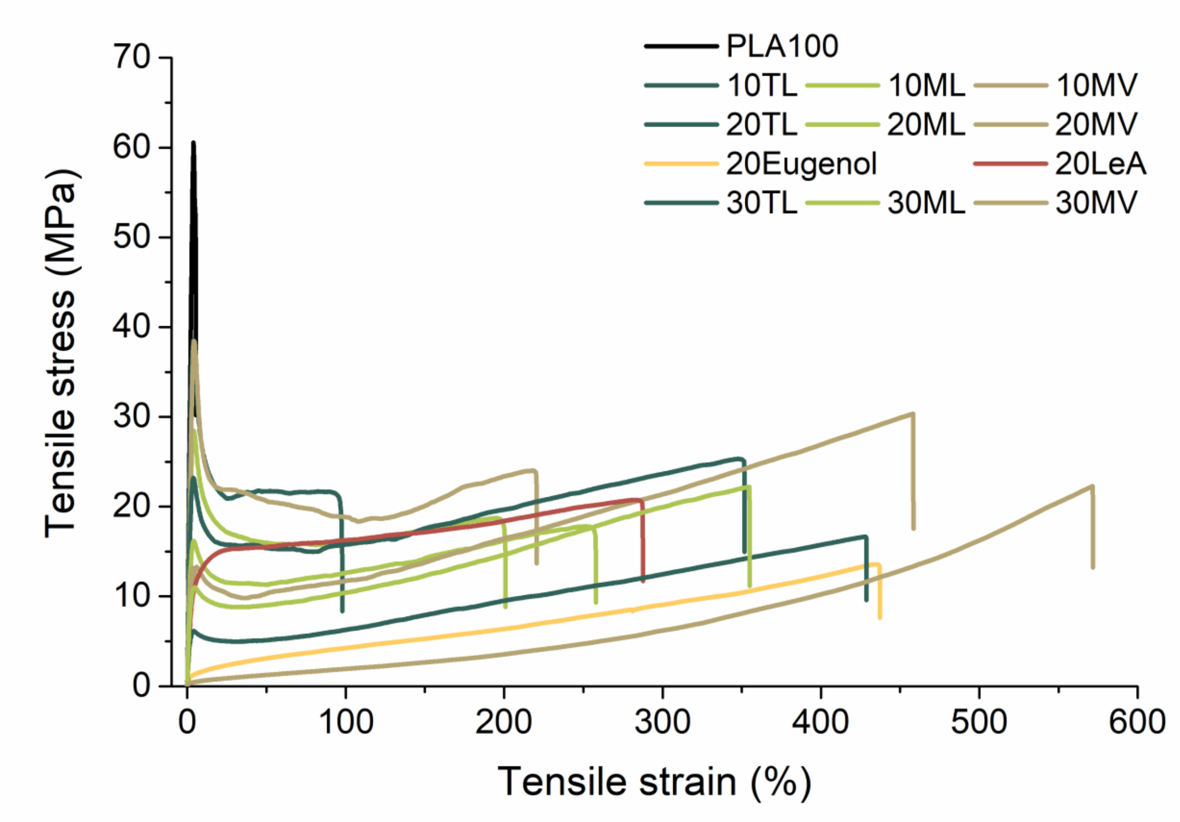

Figure S24. Representative stress-strain curves of neat PLA and its blends.

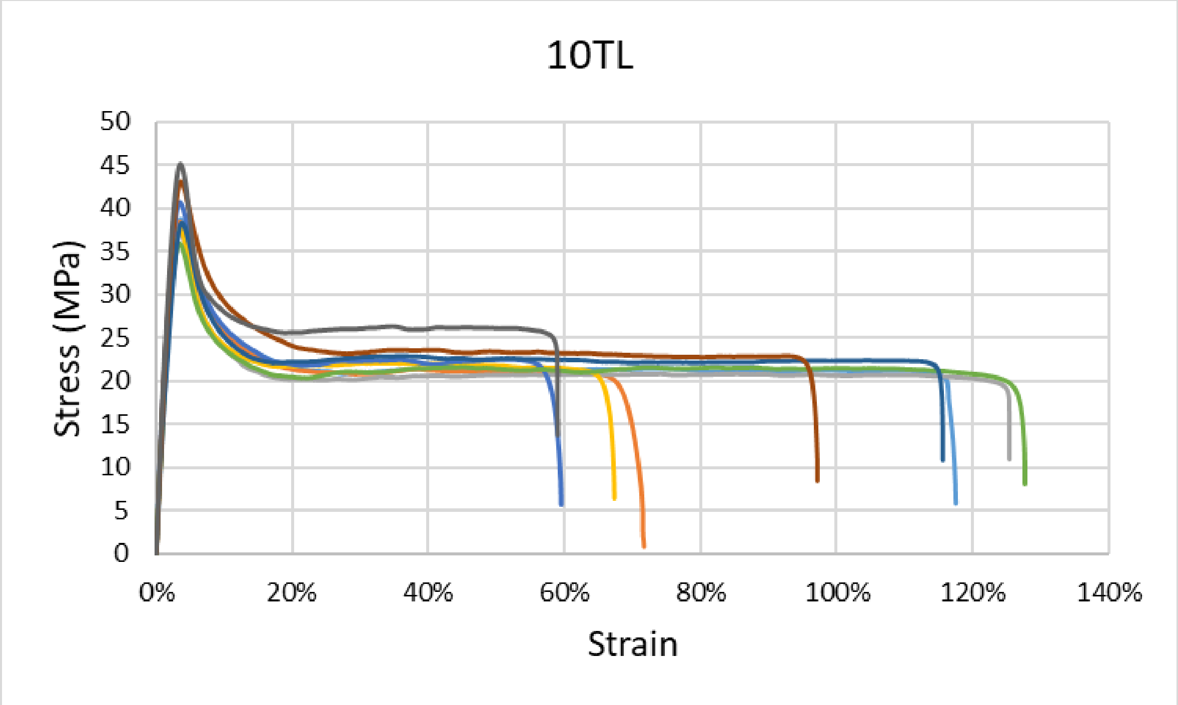

Figure S25. The tensile stress-strain curve of 10TL.

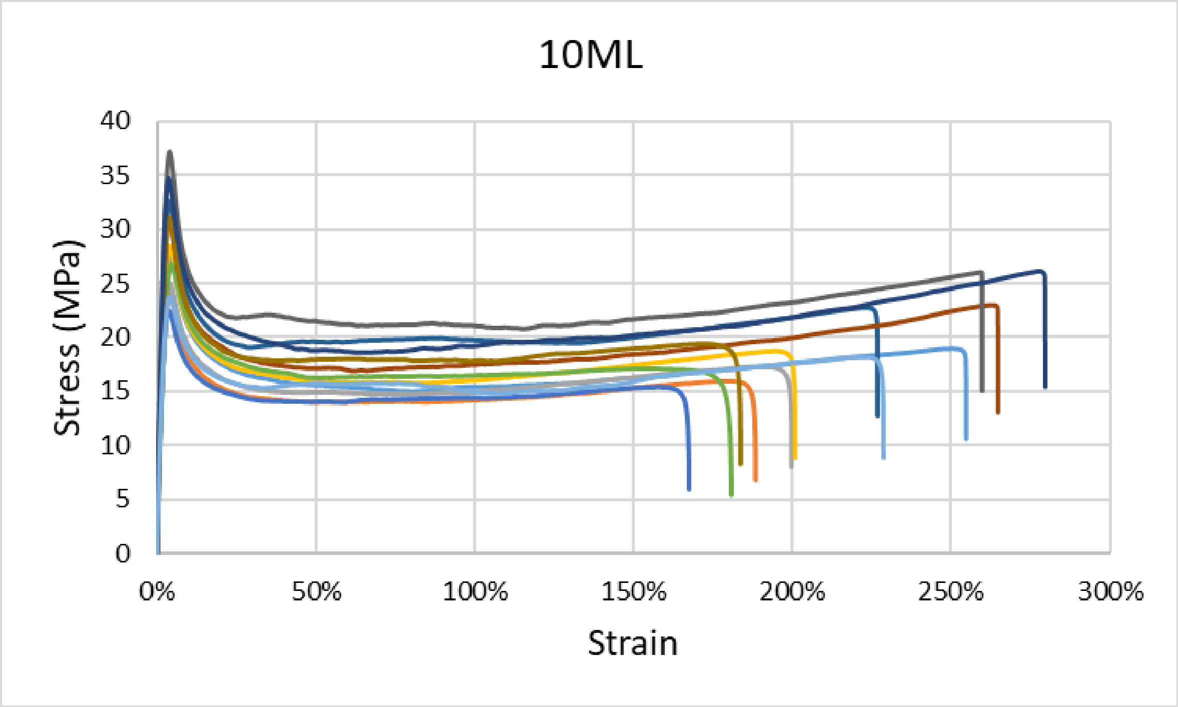

Figure S26. The tensile stress-strain curve of 10ML.

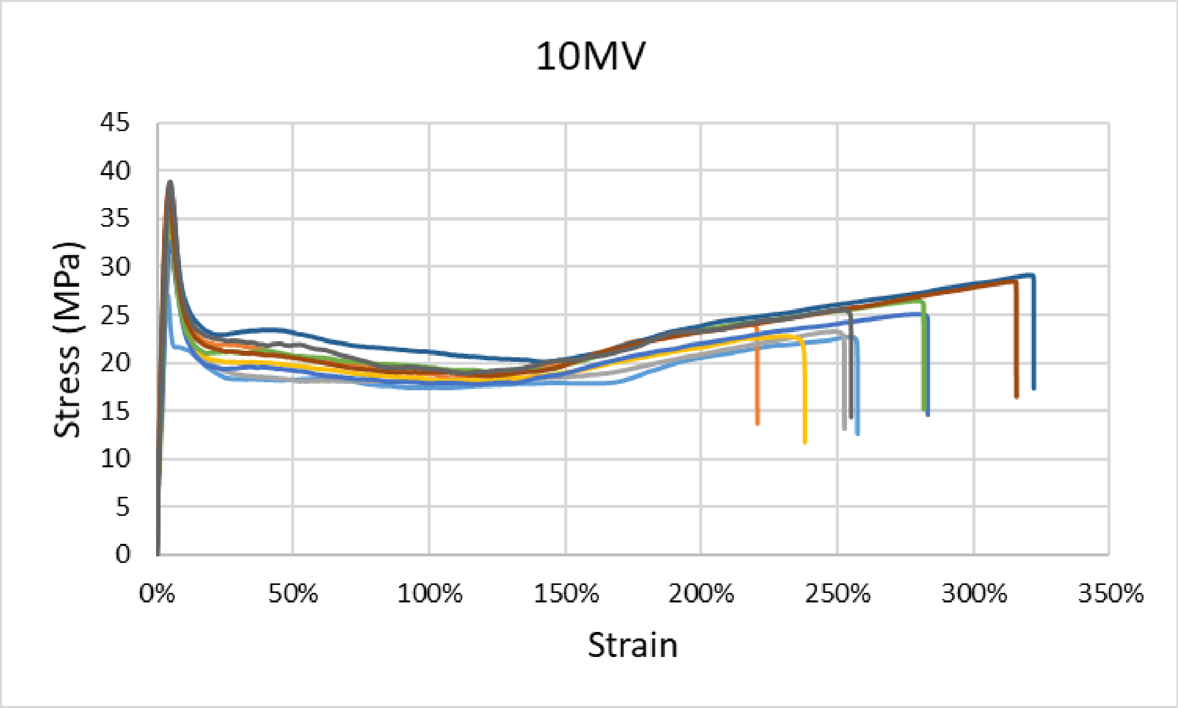

Figure S27. The tensile stress-strain curve of 10MV.

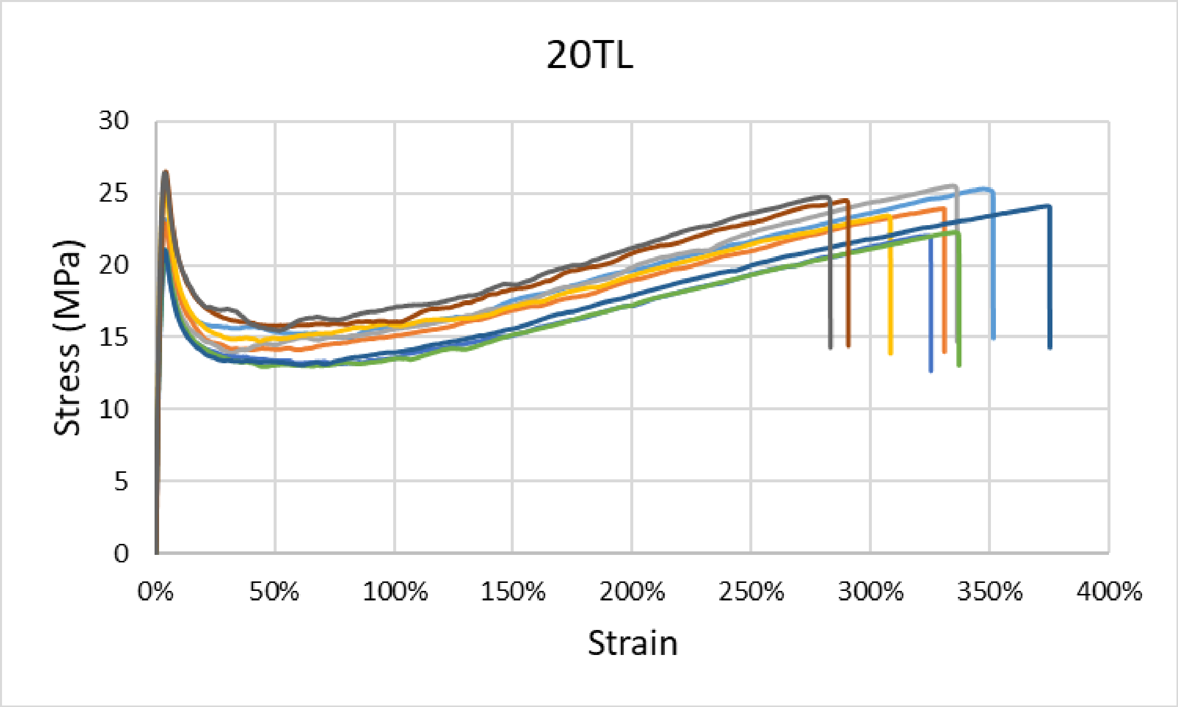

Figure S28. The tensile stress-strain curve of 20TL.

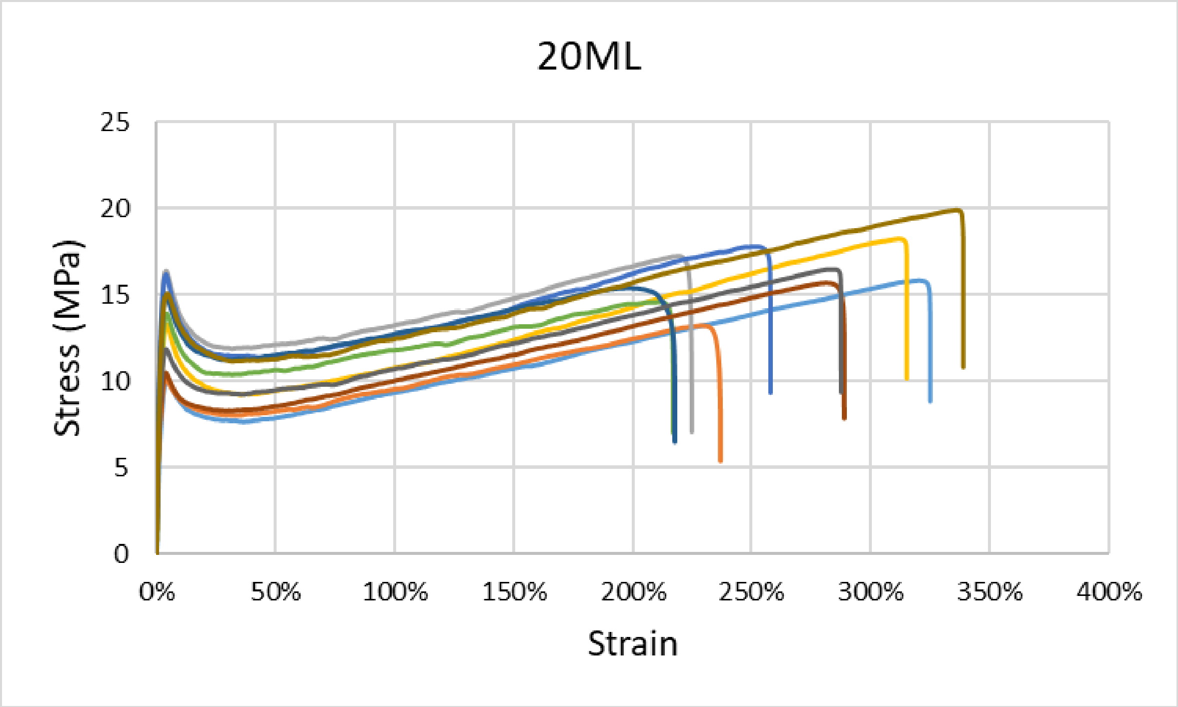

Figure S29. The tensile stress-strain curve of 20ML.

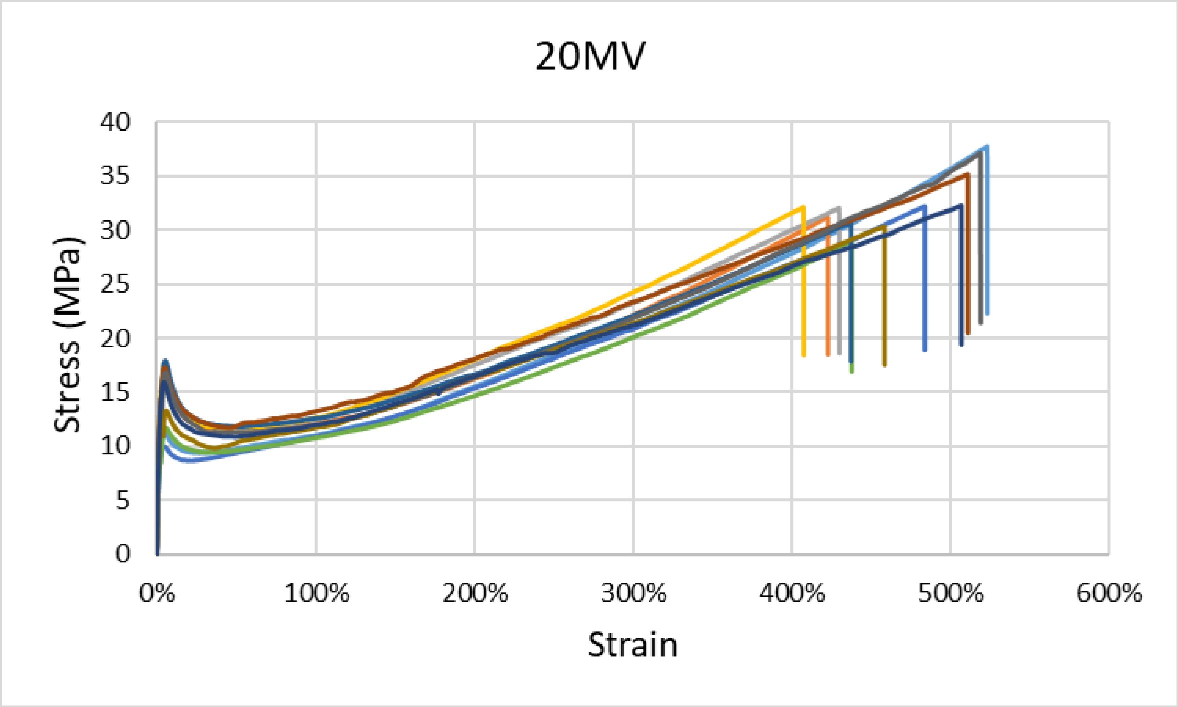

Figure S30. The tensile stress-strain curve of 20MV.

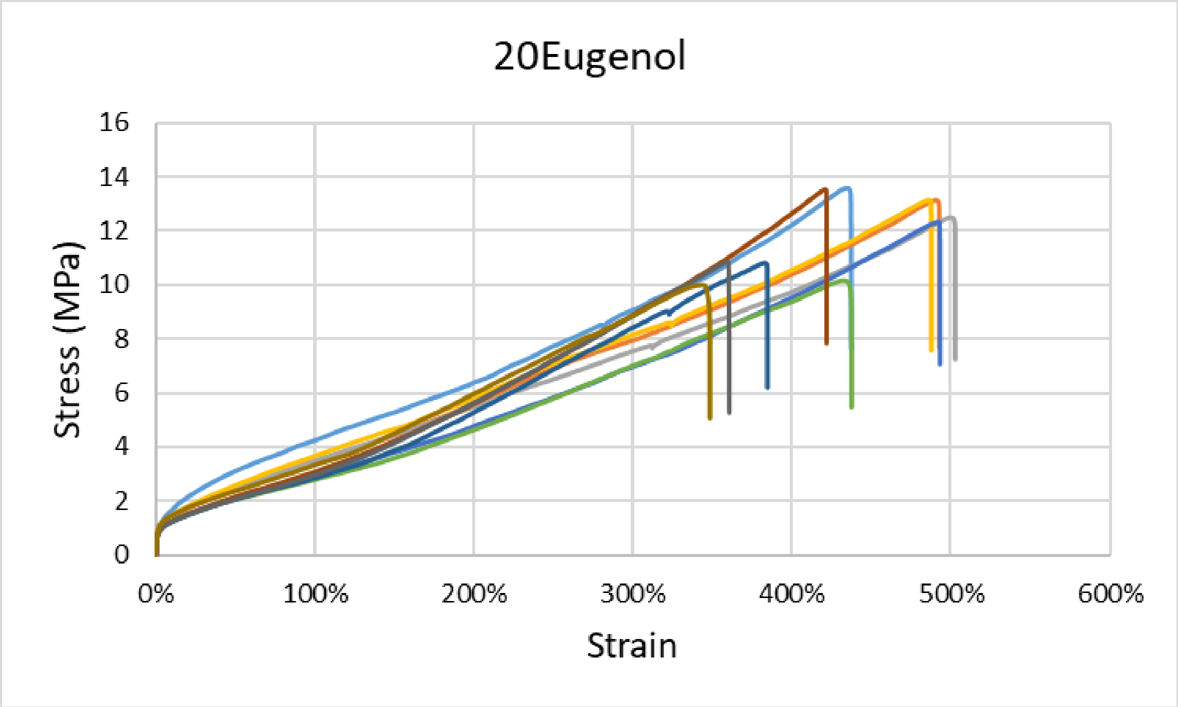

Figure S31. The tensile stress-strain curve of 20eugenol.

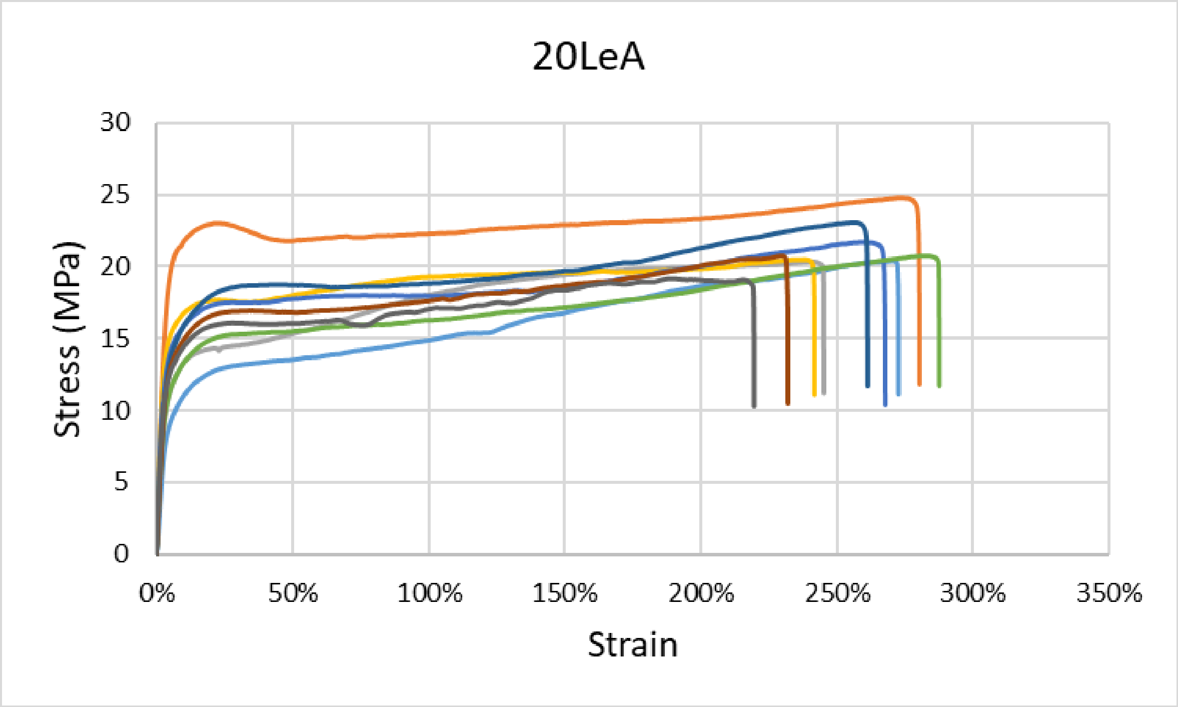

Figure S32. The tensile stress-strain curve of 20LeA.

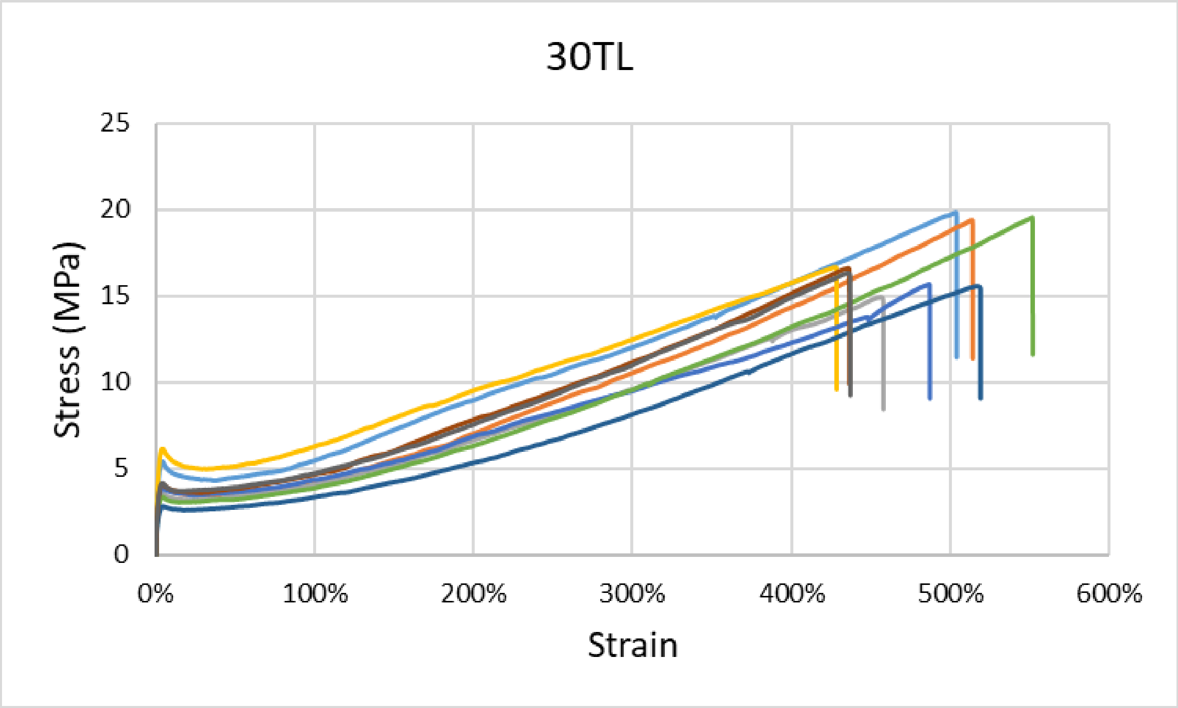

Figure S33. The tensile stress-strain curve of 30TL.

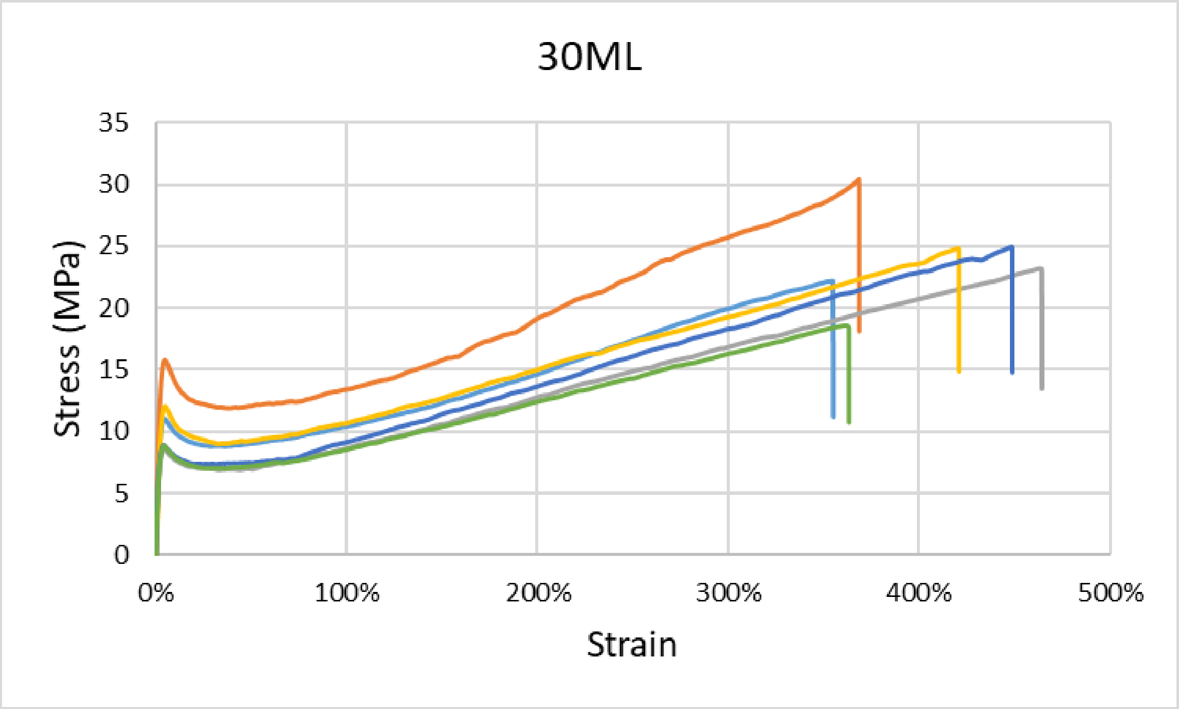

Figure S34. The tensile stress-strain curve of 30ML.

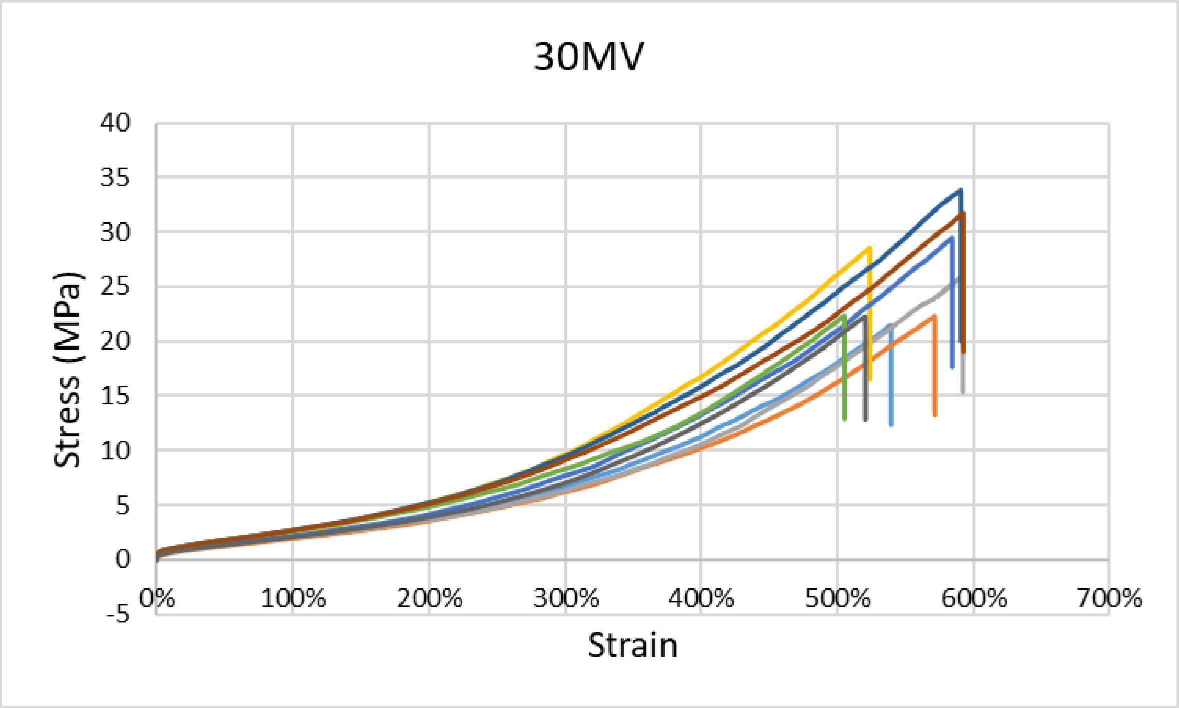

Figure S35. The tensile stress-strain curve of 30MV.

Table S10. The tensile test of neat PLA and its blends with plasticizer candidates.

	Young's Modulus (GPa)	Strain at Break (%)	Stress at Break (MPa)
PLA100	2.1 ± 0.18	5.4 ± 1.1	57 ± 6.9
10TL	1.9 ± 0.10	91 ± 28	21 ± 1.3
10ML	1.5 ± 0.26	220 ± 40	21 ± 4.1
10MV	1.3 ± 0.13	270 ± 32	25 ± 2.4
20TL	1.4 ± 0.10	330 ± 27	24 ± 1.2
20ML	0.83 ± 0.16	260 ± 44	16 ± 1.9
20MV	0.89 ± 0.13	470 ± 41	33 ± 2.7
20Eugenol	0.11 ± 0.01	440 ± 58	12 ± 1.4
20LeA	0.49 ± 0.11	250 ± 21	21 ± 1.6
30TL	0.33 ± 0.08	480 ± 41	17 ± 1.8
30ML	0.61 ± 0.08	400 ± 43	24 ± 3.6
30MV	0.06 ± 0.01	560 ± 35	26 ± 4.5

Note: The data of PLA100 were from previous work [2].

Antibacterial Study of Three Synthesized Plasticizers

Table S11. Gap distances between the plasticizer droplets and inhibition zones.

Strain \ Plasticizer	TL	ML	MV
E. coli	2.39 ± 0.46 mm	N/A	N/A
S. aureus	6.11 ± 0.33 mm	3.66 ± 0.48 mm	2.07 ± 0.56 mm

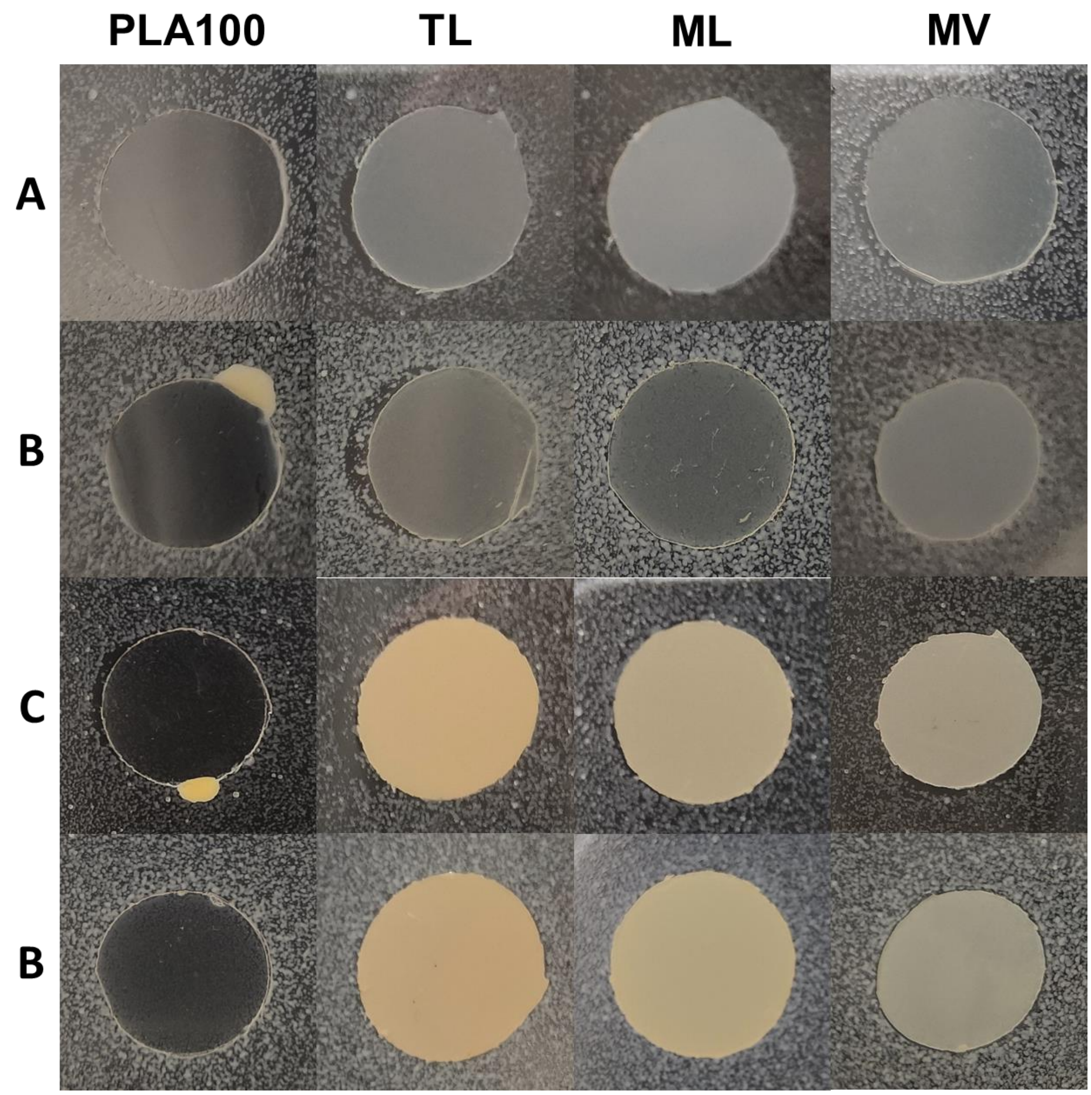

Figure S36. Zone of inhibition test of PLA and its blends with plasticizers (A: 10 wt% plasticizers, *E. coli*; B: 10 wt% plasticizers, *S. aureus*; C: 20 wt% plasticizers, *E. coli*; D: 20 wt% plasticizers, *S. aureus*).

Table S12. Diameters of inhibition zones (*S. aureus*) of paper disks loaded with plasticizers

Content \ Plasticizer	TL	ML	MV
1 mg Plasticizer	10.61 ± 0.41 mm	N/A	10.36 ± 0.46 mm
5 mg Plasticizer	12.29 ± 0.78 mm	10.66 ± 0.69 mm	12.21 ± 0.95 mm

Reference

1. Bader, A.R.; Kontowicz, A.D. γ,γ-Bis-(p-hydroxyphenyl)-valeric Acid. *J. Am. Chem. Soc.* **1954**, *76*, 4465–4466, doi:10.1021/ja01646a053.

2. Xuan, W.; Hakkarainen, M.; Odelius, K. Levulinic Acid as a Versatile Building Block for Plasticizer Design. *ACS Sustain. Chem. Eng.* **2019**, *7*, 12552–12562, doi:10.1021/acssuschemeng.9b02439.
